# Genetic Dissection of Wheat Heterosis Identifies Candidate Genes in Biparental and Diverse Hybrid Populations

**DOI:** 10.1111/pbi.70748

**Published:** 2026-08-30

**Authors:** Guoliang Li, Jianting Chu, Max Haupt, Yong Jiang, Jochen C. Reif

**Affiliations:** ^1^ Leibniz Institute of Plant Genetics and Crop Plant Research (IPK) OT Gatersleben Seeland Germany

**Keywords:** epistasis, heterosis, hQTL, hybrid, wheat

## Abstract

Understanding how numerous heterotic quantitative trait loci (hQTL) shape heterosis and shifting from hQTL to candidate genes are central challenges. To address these challenges, we performed a systematic, genome‐wide mapping in a biparental population and a population of diverse hybrids. Leveraging both a triple testcross and immortalized F_2_ designs, we examined the genetic basis of the heterosis of the wheat hybrid *Piko* × *Hermann*. We detected a major hQTL on chromosome 4B, primarily driven by epistasis. This region contains the *Green Revolution* gene *Rht‐B1* as the primary candidate gene. Then, we used a population of ~6000 wheat hybrids, derived from crosses between diverse Central European inbred lines, to perform a one‐dimensional scan for hQTL. This scan identified 174 hQTL for grain yield, 70 for heading date, and 166 for plant height. Further dissection of these hQTL revealed that epistatic interactions predominantly contribute to heterosis. We discovered an epistatic hub at the distal end of chromosome 4A coinciding with an alien introgression region from emmer wheat. A data‐driven, integrative analysis combining high‐resolution SNP data, sequence variant annotation, and hQTL signals from the population uncovered *TraesCS7B03G1341000* as the candidate gene underlying grain yield heterosis. Our findings deepen the understanding of the genetic architecture of heterosis in wheat by shifting from hQTL to candidate genes and provide insights into their application in hybrid wheat breeding.

## 1 Introduction

Midparent heterosis (MPH, hereafter referred to as heterosis) refers to the superior performance of hybrids compared to their parental lines. MPH calculated as the difference between the F_1_ hybrid performance and the mean performance of its two parents is one of the most widely used quantitative measures of heterosis (Falconer and Mackay 1996). Exploiting heterosis has been instrumental in boosting crop productivity in agriculture (Crow 1998; Duvick 2015, 2001). Three classical quantitative genetic hypotheses have been proposed to explain the genetic basis of heterosis: dominance (Bruce 1910; Huang et al. 2016; Jones 1917; Liu et al. 2020), overdominance (Crow 1948; East 1936; Hull 1945; Wang, Hou, et al. 2023), and epistasis (Jinks and Jones 1958; Li et al. 2017; Powers 1944; Zhou et al. 2012). The three hypotheses are not mutually exclusive, as reflected in the formal definition of the heterotic effect of a QTL as a linear combination of its dominance effect and various epistatic interactions (Jiang et al. 2017). Although epistasis is attributed a crucial role in the manifestation of heterosis, there is limited experimental evidence regarding the molecular determinants that shape heterosis through epistatic interactions. One exception is a pioneering study in tomato (Soyk et al. 2017), in which negative epistasis affecting yield was identified between two closely related MADS‐box transcription factors. This insight enabled researchers to overcome the observed negative epistasis through natural and gene‐edited mutations. The otherwise low success rate of identifying epistatic genes as a driving factor of heterosis in other plant species stems from significant experimental challenges in searching for epistatic genes. These genes mostly have small effects and require large population sizes for reliable detection (Wei et al. 2024).

The genetic basis of heterosis can be investigated by mapping the QTL underlying the phenotypes of a specific hybrid or populations of hybrids derived from crosses among diverse parental lines. These QTL are referred to as heterotic QTL (hQTL). For a specific hybrid, various mating designs based on biparental populations—generated by selfing the single‐cross hybrid—have been proposed and applied. These designs include segregating F_2_ populations (Huang et al. 2016; Lu et al. 2003), North Carolina Design III (NCIII, Comstock et al. 1952), triple testcross design (TTC, Kearsey and Jinks 1968; Melchinger et al. 2008), and immortalized heterozygous populations like the immortalized F_2_ (IMF_2_) and immortalized backcrosses following the concept of NCIII or TTC designs (Hua et al. 2003; Huo et al. 2023; Zhu et al. 2016). The designs leverage a well‐established quantitative genetic framework that defines the genetic effects contributing to heterosis (Cockerham and Zeng 1996; Melchinger et al. 2007), each offering distinct advantages and limitations. For instance, the NCIII and TTC designs are well‐suited for detecting the aggregated heterotic effect of a QTL, whereas the IMF_2_ design is particularly effective for identifying dominance effects. Unlike NCIII, the TTC design also enables the testing of individual epistatic interactions contributing to heterosis. Nonetheless, an integrated approach combining the advantages of NCIII/TTC and IMF_2_ designs has yet to be implemented.

Heterosis in diverse hybrid populations has been investigated using multi‐parent (Gu et al. 2023; Xiao et al. 2021) and factorial mating designs (Gu et al. 2023; Jiang et al. 2017). Individual components of heterosis can be identified and, in some cases, integrated to assess the aggregated heterotic effect of a QTL (Jiang et al. 2017). Interestingly, many experimental studies on crops such as rice, maize, and cotton primarily focus on dominance (Lin et al. 2020; Liu et al. 2020) and overdominance effects (Gu et al. 2023; Shang et al. 2016), and only a few consider epistasis (Li et al. 2018; Sang et al. 2022). The emphasis on the former is largely due to the high computational demands of testing the vast number of potential epistatic interactions, which is especially challenging in deeply sequenced populations (Liu et al. 2020). Furthermore, the proposed two‐step procedure (Jiang et al. 2017) often lacks the sensitivity to detect hQTLs that are composed of numerous components with small individual effects, which, when combined, make a substantial contribution to heterosis (Li et al. 2025). These limitations were addressed in a recent study that introduced a one‐dimensional genome‐wide scan for hQTLs (hQTL‐ODS). This approach revealed widespread, cumulative epistatic effects that contribute to heterosis (Li et al. 2025). The study did not provide a deep and comprehensive analysis of the hQTL regions, their putative underlying genes and hQTL genetic interactions because the primary focus was on investigating the methodology.

In this study, we integrated analyses from both a biparental wheat population and a large, diverse hybrid population to obtain a comprehensive view of the genetic architecture underlying wheat heterosis (Figure 1). We first combined the complementary TTC and IMF_2_ designs (Figure 1b) in a biparental population, enabling the identification of a major hQTL on chromosome 4B associated with grain yield heterosis. We then applied the novel hQTL‐ODS approach (Li et al. 2025) to a comprehensive hybrid wheat population comprising 5243 hybrids derived from crosses among deeply sequenced and genetically diverse inbred lines (Figure 1c). This analysis revealed a prominent epistatic hub on chromosome 4A that colocalized with an alien introgression from emmer wheat, as well as a key candidate gene, *TraesCS7B03G1341000*, encoding a grain‐preferential NAC transcription factor. These findings not only advance our understanding of the genetic basis of heterosis in wheat but also provide potential genetic targets for future breeding programs.

**FIGURE 1:**
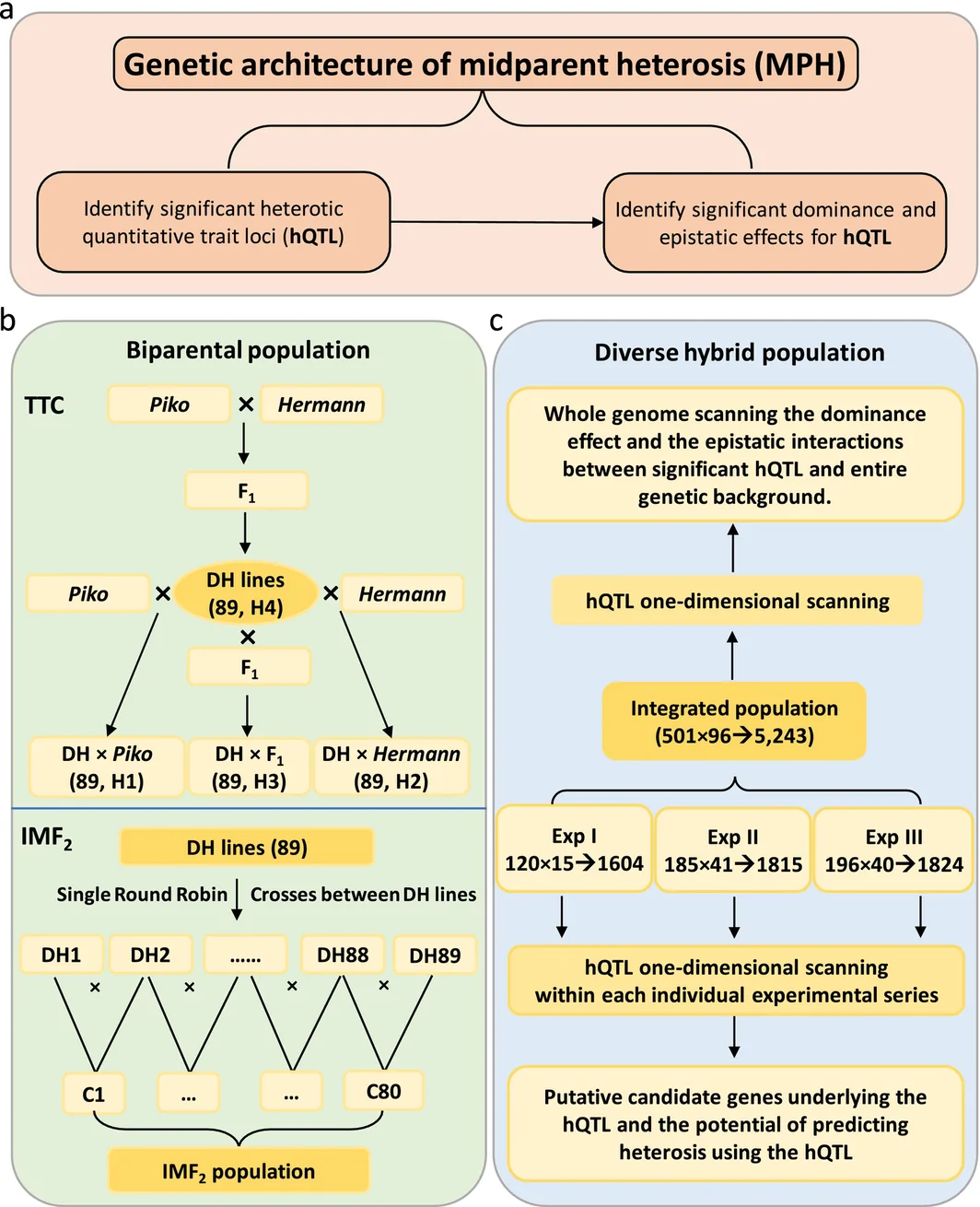
The experimental design, analysis procedure and materials used in the study. (a) Procedure to dissect the genetic architecture of heterosis. (b) Outline of the procedure to test heterotic effects in biparental population, including both triple testcross (TTC) design and immortalized F_2_ (IMF_2_) design. (c) Outline of the procedure to test heterotic effects in diverse hybrids population using hQTL‐ODS approach.

## 2 Results

### 2.1 A Major hQTL on Chromosome 4B Functions as an Epistatic Hub of Grain Yield Heterosis in *Piko* × *Hermann*

We implemented both the IMF_2_ and TTC designs for a population of doubled haploid lines (DHs) of the *Piko* × *Hermann* hybrid. The quality of the phenotypic data was high, with heritability estimates ranging from 0.61 for grain yield to 0.90 for plant height in the IMF_2_ design, and from 0.82 for plant height to 0.92 for grain yield in the TTC design (Table S1). Relative midparent heterosis was highest for grain yield at 8.8%, followed by plant height at 7.6%, and lowest for heading date at −0.5% (Table S1). The highest relative better parent heterosis (BPH) was for grain yield at 7.84%, followed by plant height at 6.34%, and for heading date at −0.8% (Table S1). Positive BPH for grain yield was observed in four of the six environments with available phenotypic data, illustrating that the heterotic advantage was maintained under most environments despite substantial variation in absolute grain yield (Figure S1).

The TTC design provides valuable insights into the role of epistasis by comparing the performance of DHs when crossed with *Piko* and *Hermann* with the performance of DHs when crossed with the *Piko* × *Hermann* hybrid (Melchinger et al. 2008). This phenotypic‐based test was significant for all analysed traits in our study (Table S2). Consequently, even at this aggregated level of analysis, the contribution of epistasis to the heterosis of the *Piko* × *Hermann* hybrid is evident.

QTL mapping based on the performance differences between DHs when crossed with *Piko* and *Hermann* directly assesses the hQTL effect in a one‐dimensional scan (Melchinger et al. 2008). This scan identified a single hQTL on chromosome 4B for both grain yield and plant height (Figure 2a,e), as well as two hQTL on chromosome 2B and 7D for heading date (Figure S2a). Notably, the hQTL region on chromosome 4B encompasses the *Rht‐B1* gene (Peng et al. 1999) and explains 15.2% of the phenotypic variation in heterosis for grain yield and 6.2% for plant height.

**FIGURE 2:**
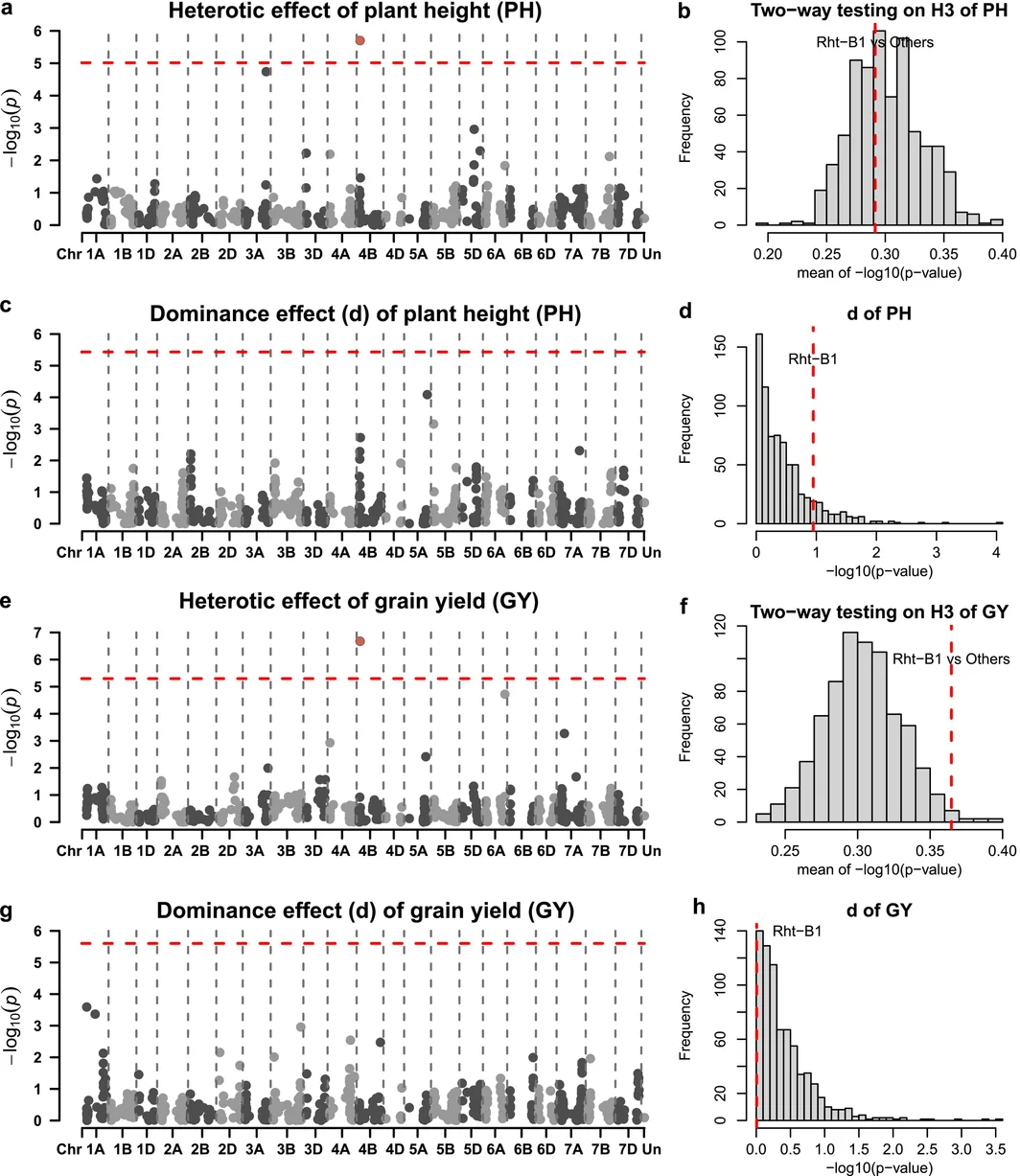
Genetic architecture of midparent heterosis for plant height (PH, cm) and grain yield (GY, Mg/ha) in the hybrid *Piko* × *Hermann*. The genome wide scan of hQTL within the TTC population for plant height (a) and grain yield (e). Two‐way marker interactions detected in the performance of DH × (*Piko* × *Hermann*) (H3) indicate the epistatic component of the heterotic effect, shown by the distribution of –log10(*p*‐value) for plant height (b) and grain yield (f), where the red dashed vertical line indicates the mean of –log10(*p*‐value) between hQTL (*Rht‐B1*) and all other markers. A genome‐wide scan of the dominance effect for plant height (c) and grain yield (g) was conducted within IMF_2_ population, with the distribution of –log10(*p*‐value) shown for PH (d) and GY (h), where the red dashed vertical line indicates the –log10(*p*‐value) of hQTL (*Rht‐B1*). The red dashed horizontal line in a, c, e, and g indicates the significance threshold (*p* < 0.05) determined from 1000 permutation tests.

We next investigated whether the detected hQTL are primarily driven by dominance effects or by additive‐by‐additive epistatic interactions. The IMF_2_ design enabled us to specifically assess the contribution of dominance effects to heterosis (Hua et al. 2003). Interestingly, we did not detect significant dominance effects for any of the three traits (Figure 2c,g, Figure S2b), suggesting that dominance effects are not major contributors to heterosis in the *Piko* × *Hermann* hybrid, which indirectly indicates that epistasis may play an important role.

To directly test this hypothesis, we analysed the performance of crosses between DH lines and the *Piko* × *Hermann* hybrid, enabling the detection of additive‐by‐additive epistatic effects through a two‐dimensional genome‐wide scan (Melchinger et al. 2008). For grain yield, five pairs of markers showed significant additive‐by‐additive interactions between chromosome 1A and 4B at a 5% false discovery rate (Table S3). Interestingly, all five markers involved on chromosome 4B were in proximity to the identified hQTL, with a genetic distance of 17 cM. We then calculated the average ‐log(*p*) values of the interaction effects between each marker and all other markers. For grain yield, the leading marker located in the hQTL region showed substantially higher average ‐log(*p*) values than most markers elsewhere on the genome (Figure 2f), indicating consistent epistatic signals centred on the hQTL region. Combined with the minimal contribution of dominance effects to grain yield heterosis (Figure 2h), these findings support the conclusion that epistatic interactions involving the hQTL on chromosome 4B play a central role in mediating grain yield heterosis. On the other hand, for plant height, the mean ‐log(*p*) values of the interaction effects between the hQTL and the genetic background were only average (Figure 2b), and the dominance effect of the hQTL was stronger than many other markers (Figure 2d), but not yet reached the genome‐wide threshold (Figure 2c). These results indicate that the contribution of the hQTL on chromosome 4B to plant height heterosis is likely due to mild dominance effect together with cumulative small epistatic effects.

The hQTL for grain yield heterosis on chromosome 4B encompassed *Rht‐B1*, but also many additional genes. To investigate the candidate genes within this region, whole‐genome resequencing data from *Piko* and *Hermann* were analysed to explore genetic variation across the hQTL interval in detail. The significant bin marker TG0010b (Chr4B:33614738) was flanked by SNP markers AX‐158537145 and AX‐158583109, located at positions 30 610 615 bp and 43 391 027 bp, respectively, defining a 12.78 Mb interval.

SNPs within this region were filtered to retain only those polymorphisms between *Piko* and *Hermann*, resulting in 20 443 variants, with an average read depth of 3.32 for *Piko* and 2.56 for *Hermann*. All genes within this interval were extracted, and genetic variant annotation was performed using ANNOVAR (Wang et al. 2010), with 1000 bp windows applied to define upstream and downstream gene regions. This analysis identified 279 SNPs in gene‐associated regions differentiating the two parental lines. Among these, 16 genes harboured nonsynonymous sequence variants, including *TraesCS4B03G0093100* (*Rht‐B1*). In addition, 32 genes contained SNPs located in 3′UTRs, 5′UTRs, or within the upstream/downstream flanking regions (Table S4). To further prioritize candidate genes, functional information of all 48 genes was retrieved from the Triticeae‐Gene Tribe database (http://wheat.cau.edu.cn/TGT/; Chen et al. 2020), which provides gene models for various Chinese Spring genome versions, orthology relationships with *Arabidopsis thaliana* , and functional annotations. Functional annotations for Arabidopsis homologues were obtained from The Arabidopsis Information Resource (TAIR; https://www.arabidopsis.org/). Among the 48 genes, six genes, including *TraesCS4B03G0093100* (*Rht‐B1*), were prioritized as the high‐confidence candidate genes because they were identified as RBHs to *Arabidopsis thaliana* genes and harboured nonsynonymous sequence variants (Table S4). Although we also examined the expression profiles of all 48 genes using published developmental time‐course data for the wheat genotype Chinese Spring (Choulet et al. 2014), no additional evidence was obtained to further refine the candidate gene list.

### 2.2 Epistasis Drives Wheat Heterosis in Diverse Central European Hybrids

While mapping hQTL in biparental populations optimizes the power to detect certain genetic effects, limitations exist in several aspects; their utility is limited by dependence on parental selection and low mapping resolution due to extensive linkage disequilibrium. Association mapping in diverse populations therefore provides a powerful complementary approach. Results from a large wheat population of 5243 hybrids derived from crosses among 597 Central European parental lines revealed multiple hQTL for grain yield and heading date (Li et al. 2025). A targeted reanalysis of these data holds the potential to provide further insights into the widespread presence of epistasis contributing to heterosis in wheat. Compared with the previous analysis (Li et al. 2025), the markers used in the present study were refined through LD‐based inspection and filtering (see Section 4), enabling more precise candidate gene characterization in downstream analyses. In addition to summarizing the previous findings on grain yield and heading date, we expanded the analysis to include plant height. In the study, the wheat hybrids and their parental lines were phenotyped across three experimental series (Exp I, II, and III, Figure 1c and Figure S3), with each trait evaluated in over 10 environments within each series (Table S5). These three experimental series were subsequently combined into a single integrated population (Figure 1c). The excellent quality of the phenotypic data was demonstrated by the high broad‐sense heritability of both trait performance and heterosis (Table S5). In the integrated population, the heritability estimates of plant height were 0.98, 0.95, and 0.87 for the performance of hybrids, parents, and MPH, respectively (Table S5).

We used the hQTL‐ODS method (Li et al. 2025) to analyse the genetic architecture of heterosis in wheat across the integrated population and the three experimental series. Whole‐genome resequencing provided ~0.9 million high‐quality SNPs in the integrated population (Figure S3). A total of 3034 SNPs associated with grain yield heterosis grouped into 174 hQTL (see Supporting Information for details on the delineation of QTL from significant SNPs), explaining 1.21% to 16.84% of phenotypic variance (Table S6, Figure 3a). The most significant hQTL were on chromosomes 7B (*Integrated_GY_hQTL167*, explained 11.18% variance) and 4A (*Integrated_GY_hQTL121*, 12.62% variance), while the phenotypic variance explained (PVE) by *Integrated_GY_hQTL142* on chromosome 6B was the largest (16.84%). Only three dominance QTL (dQTL) co‐localized with hQTL, contributing minimally (0.68%–0.76%) to heterosis variance, suggesting epistasis as the main driver of grain yield heterosis (Figure 3, Table S7).

**FIGURE 3:**
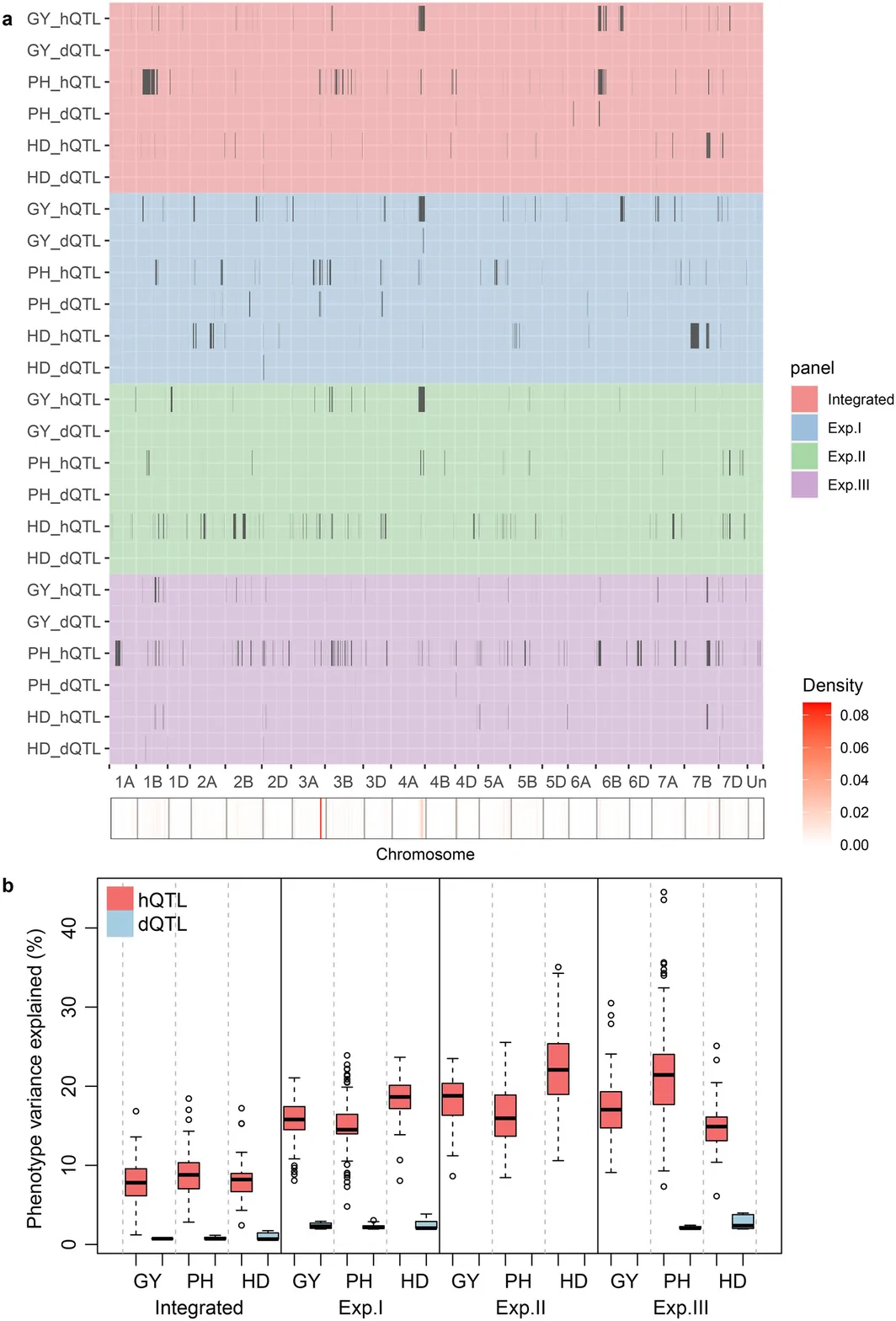
Genetic architecture of midparent heterosis (MPH) in wheat. (a) All heterotic QTL (hQTL) and dominance QTL (dQTL) detected in the integrated population and within each individual experimental series for grain yield (GY), plant height (PH), and heading date (HD). (b) The phenotypic variance explained (%) by hQTL and dQTL.

Similar patterns were observed for heading date and plant height heterosis in the integrated population. For heading date, 499 significant SNPs grouped into 70 hQTL with PVE ranged between 2.42% and 17.22% (Table S8, Figure 3a). The most significant *hQTL Integrated_HD_hQTL026* (PVE = 8.86%) on chromosome 2D included the *Ppd‐D1* gene, while the *hQTL Integrated_HD_hQTL059* on chromosome 7B had the highest PVE (17.22%). Only five dQTL (PVE ranged between 0.65% and 1.74%) were found, and three of them overlapped with hQTL including *Ppd‐D1* (Table S9). For plant height, 166 hQTL explained 2.83% to 18.44% of variance (Table S10), with a key hQTL *Integrated_PH_hQTL082* (PVE = 11.49%) on chromosome 4D overlapped with the *Rht‐D1* gene. Of 10 dQTL (PVE ranged between 0.63% and 1.16%), four co‐localized with hQTL, including *Rht‐D1* (Table S11). Overall, these results indicate that epistatic interactions outweigh dominance effects in driving wheat heterosis for all three traits, both in terms of their abundance and their contribution to phenotypic variance, and the conclusion is consistently supported across all experimental series (Figure 3, Tables S12–S27).

### 2.3 Emmer Introgression on 4A Acts as an Epistatic Hub Driving Grain Yield Heterosis

We found that over half of the hQTL identified for grain yield (85 out of the 174) are clustered at the distal end of chromosome 4A. This region spans from 602.37 M base pairs (Mb) to 754.16 Mb and includes hQTL *Integrated_GY_hQTL037* to *Integrated_GY_hQTL121* (Table S6 and Figure S4a). Notably, *Integrated_GY_hQTL121* (706.29–754.16 Mb) encompasses an alien introgression (732.98–754.23 Mb) derived from emmer wheat and is associated with a large‐scale inversion (Przewieslik‐Allen et al. 2021; Schulthess et al. 2022). The 248 significant SNPs within this hQTL exhibited strong linkage, with an average *r* ^2^ value of 0.76 between pairs of markers and an estimated number of effective markers (Gao et al. 2008) of 1. Extending the analysis to all 2043 SNPs underlying the 89 hQTL in this region revealed tightly linked clusters (Figure S5), with an estimated effective number of markers of 3. Further examination of heterotic effects in hybrids lacking the emmer wheat introgression revealed no detectable hQTL in this region (Figure S6), suggesting that the introgression region on chromosome 4A is a key driver of heterosis.

Given that none of the 85 hQTL identified on chromosome 4A exhibited significant dominance effects (Figure 3a), we hypothesized that epistatic interactions must play an important role in composing these hQTL. To explore this, we conducted a genome‐wide analysis of digenic epistatic interaction between all 174 hQTL and the remaining genomic background. To reduce the computational load of testing a large number of pairwise interactions, 511 tagging SNPs were chosen to represent the 174 hQTL (see Section 4 for details). This analysis revealed 175 794 significant epistatic interactions (Bonferroni‐corrected threshold, *p*‐value < 0.01). These interactions were categorized into pairs of interaction regions, comprising 1138 additive‐by‐additive, 448 additive‐by‐dominance, 922 dominance‐by‐additive, and 377 dominance‐by‐dominance interaction regions (Figure 4).

**FIGURE 4:**
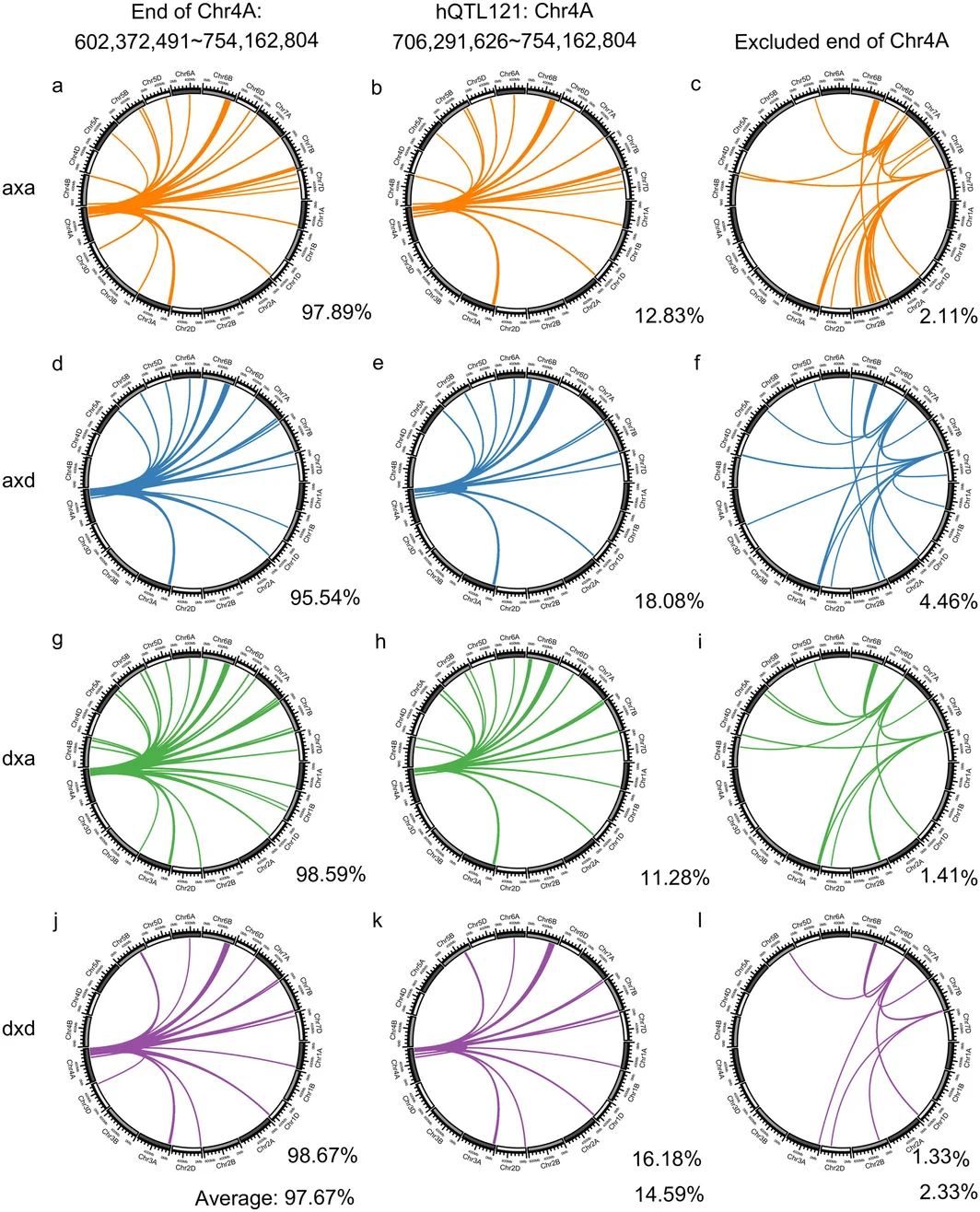
Significant digenic epistatic interactions between hQTL and the rest of the genomic background for MPH of grain yield in the integrated population. The figure is divided into three columns: first column (a, d, g, j) shows the significant epistatic interactions involving hQTL from the distal end of chromosome 4A (from 602 372 491 to 754 162 804, including *Integrated_GY_hQTL037~121*), second column (b, e, h, k) shows the significant epistatic interactions involving hQTL from *Integrated_GY_hQTL121* (from Chr4A:706 291 626 to 754 162 804) that exactly covered the introgression region (from Chr4A:732 979 379 to 754 227 511), and the third column (c, f, i, l) shows the significant epistatic interactions involving hQTL excluding those from the distal end of chromosome 4A; the epistatic interactions are categorized as (a–c) for additive‐by‐additive (a × a), (d–f) for additive‐by‐dominance (a × d), (g–i) for dominance‐by‐additive (d × a), and (j–l) for dominance‐by‐dominance effects (d × d).

Strikingly, the 85 hQTL located at the distal end of chromosome 4A accounted for an average of 97.67% (over the four categories of digenic epistatic pairs) of all significant epistatic interactions (Figure 4). Among these, 24.5% included SNPs closely associated with the introgression segment. Of particular significance, the hQTL *Integrated_GY_hQTL121*, spanning 706.29–754.16 Mb and harbouring the alien introgression, alone accounted for 14.59% of all significant interactions (Figure 4). Its tagging SNP, Chr4A_748203718, displayed the strongest interaction with the SNP Chr7B_763461403 which represents the most significant hQTL *Integrated_GY_hQTL167* on chromosome 7B (Figure S7a). In contrast to the hQTL *Integrated_GY_hQTL121* on chromosome 4A which interacts with many regions across the genome, the other important hQTL, e.g., *Integrated_GY_hQTL167* (the most significant one in terms of *p* values) on 7B interact almost exclusively with the distal end of chromosome 4A (Figure S7b). Together, these findings suggest the emmer wheat introgression on chromosome 4A acting as a major epistatic hub with a pivotal role in grain yield heterosis in wheat.

### 2.4 Co‐Localization of Grain Yield hQTL With Plant Height and Heading Date hQTL

Pleiotropic hQTL shared between grain yield and traits with higher plot‐based heritability, such as plant height or heading date, represent valuable targets for efficient map‐based cloning. We identified 74 genomic regions where grain yield hQTL overlapped with plant height hQTL (Table S28). To evaluate whether this overlap was greater than expected by chance, we performed a permutation‐based enrichment analysis with 10 000 simulations, randomly distributing hQTL intervals while preserving their number and size. The observed overlap (*n* = 74) was significantly enriched (empirical *p* = 5.99 × 10^−5^). In 20 of these regions, grain yield and plant height hQTL shared the same lead SNP. Notably, the hQTL *Integrated_GY_hQTL142* on chromosome 6B with the highest PVE for grain yield, and the most significant grain yield hQTL *Integrated_GY_hQTL167* on chromosome 7B were among the overlapping loci. In contrast, *Integrated_GY_hQTL121*, the major grain yield hQTL on chromosome 4A, did not overlap directly with any plant height hQTL. The absence of pleiotropic effects, together with limited recombination, hampers the fine‐mapping of this hQTL.

Additionally, we identified eight genomic regions where grain yield hQTL co‐localized with heading date hQTL. Using the same permutation‐based framework, we confirmed that the observed overlap (*n* = 8) was not significantly greater than expected by chance (empirical *p* = 0.118). Moreover, none of the three major grain yield hQTL (*Integrated_GY_hQTL121*, *Integrated_GY_hQTL142*, and *Integrated_GY_hQTL167*) overlapped with heading date hQTL. These findings indicate that heading date is unlikely to be a useful proxy trait for fine‐mapping the major loci underlying grain yield heterosis.

### 2.5 *TraesCS7B03G1341000* Is a Strong Candidate Gene Underlying the Grain Yield hQTL on Chromosome 7B

In the first step of nominating candidate genes, we focused on the hQTL detected in the integrated population. For grain yield heterosis, a total of 3510 genes were found within the 174 hQTL regions, including 176 genes harbouring significant SNPs (Tables S6 and S26). Among the 176 genes, 53 contain nonsynonymous variants, splice donor or acceptor site variants, and stop‐gain/loss mutations, which are more likely to affect protein function (Figure S3, Table S26). Notably, each of the two potentially pleiotropic major hQTL *Integrated_GY_hQTL142* (6B) and *Integrated_GY_hQTL167* (7B) harbours three genes carrying such functional variants, whereas the epistatic major hQTL *Integrated_GY_hQTL121* (4A) contains 35 such genes.

In order to further narrow down the candidate genes, we implemented a data‐driven approach incorporating the analysis for single environments in individual experimental series (Figure S8), similar to the recently developed framework (Wang et al. 2026). Briefly, the significance of all SNPs in the hQTL regions, including those previously excluded during LD pruning, was re‐evaluated at two hierarchical levels: across‐environment within each of the three experimental series and within each environment of each series. Then, SNPs repeatedly detected to show significant heterotic effects (hSNPs) and predicted to have functional impacts were considered the most informative variants for prioritizing candidate genes. More precisely, two categories of hSNPs were considered: (i) hSNPs detected in all three experimental series (cross‐series hSNPs, cs‐hSNPs), and (ii) hSNPs identified in at least two environments regardless of whether they occurred within or across experimental series (multi‐environment hSNPs, me‐hSNPs).

For grain yield heterosis, we only identified nine cs‐hSNPs, all clustered within a region on chromosome 7B spanning 762 665 657–763 530 826 bp and collocated with the hQTL *Integrated_GY_hQTL167* (Table S29). Among these nine cs‐hSNPs, six were related to the gene *TraesCS7B03G1341000* (*TaGNAC1‐7B*). Notably, the cs‐hSNP Chr7B_763527707 was located within an exon region and was predicted to be a nonsynonymous variant, whereas the remaining five cs‐hSNPs were located downstream of the gene, including two within 1 kb and three within 3 kb (Table S29). Thus, the gene *TraesCS7B03G1341000* is prioritized as a strong candidate gene for grain yield heterosis. It encodes a grain‐preferential NAC transcription factor (GNAC, for ‘Grain‐NAC’) that is highly expressed during the grain cell division stage. Its homologous gene, wheat *NAC‐A18* (*TraesCS7A03G1388600*), has been reported to regulate grain starch and storage protein synthesis and to affect grain weight (Wang, Liu, et al. 2023). Switching to me‐hSNPs, we identified 2565 me‐hSNPs on chromosome 4A, which allowed us to reduce the number of candidate genes for the hQTL *Integrated_GY_hQTL121* from 35 to 24 (Table S30). It would be quite challenging to further reduce the number because of the suppressed recombination rate of the alien introgression region. In contrast, 22 me‐hSNPs were concentrated in a distal chromosome 7B region spanning 746 827 575–763 530 826 bp. Functional annotation of these variants prioritized two candidate genes, *TraesCS7B03G1340100* and *TraesCS7B03G1341000* (Table S30). Notably, the latter was independently identified as an important candidate gene again.

Similar approaches were applied to the other two traits. For plant height heterosis, 4285 genes were identified within 166 hQTL, among which 70 genes carried significant SNPs and 14 contained nonsynonymous variants (Tables S10 and S27). For heading date heterosis, 1468 genes were located within 70 hQTL, including 12 genes harbouring significant SNPs and 4 that contained nonsynonymous variants (Tables S8 and S27). No cs‐hSNPs were detected for both traits (Tables S31 and S33), whereas 11 and 5 me‐hSNPs were identified for heading date and plant height heterosis, respectively. Although no candidate gene could be directly nominated, *Ppd‐D1* was located only 85.276 kb from the nearest me‐hSNP for heading date heterosis (Table S32). Likewise, *Rht‐D1* was approximately 350 kb away from the nearest me‐hSNP for plant height heterosis (Table S34).

### 2.6 Exploiting hQTL in Hybrid Wheat Breeding

The prominent role of epistasis in the genetic architecture of heterosis revealed by our findings suggests that hybrid wheat breeding should prioritize specific allele combinations across loci rather than focusing solely on individual markers. To illustrate this point, we not only compared MPH patterns associated with the two most significant hSNPs and their allelic combinations (Figure 5b–d), but also selected 10 loci across seven different chromosomes that showed the most significant epistatic interactions with the peak SNP Chr4A_748203718 representing *Integrated_GY_hQTL121* (Figure 5a,e). Theoretically, these 10 loci can form 3^10^ = 59 049 possible allele combinations. However, only 23 allele combinations were represented by more than 30 hybrids in the integrated population. A two‐way analysis of variance (ANOVA) modelling the allele combinations at the 10 loci (23 levels), the genotype at Chr4A_748203718 (three levels) and their interactions revealed that the allele combinations defined by these 10 loci had a highly significant effect on MPH (*p* < 2.0e−16, Table S35), and their interaction with Chr4A_748203718 was also highly significant (*p* = 7.54e−16, Table S35). Despite this, no significant differences in MPH were observed when comparing the allele classes of Chr4A_748203718 alone (Figure 5b and Table S35). Therefore, the allele status at the 4A locus alone is not helpful for identifying hybrids with superior heterosis. Instead, taking the 10 loci significantly interacting with the locus into account leads to some favourable allele combinations, which may possibly be considered as ‘ideotypes’ (Figure 5e).

**FIGURE 5:**
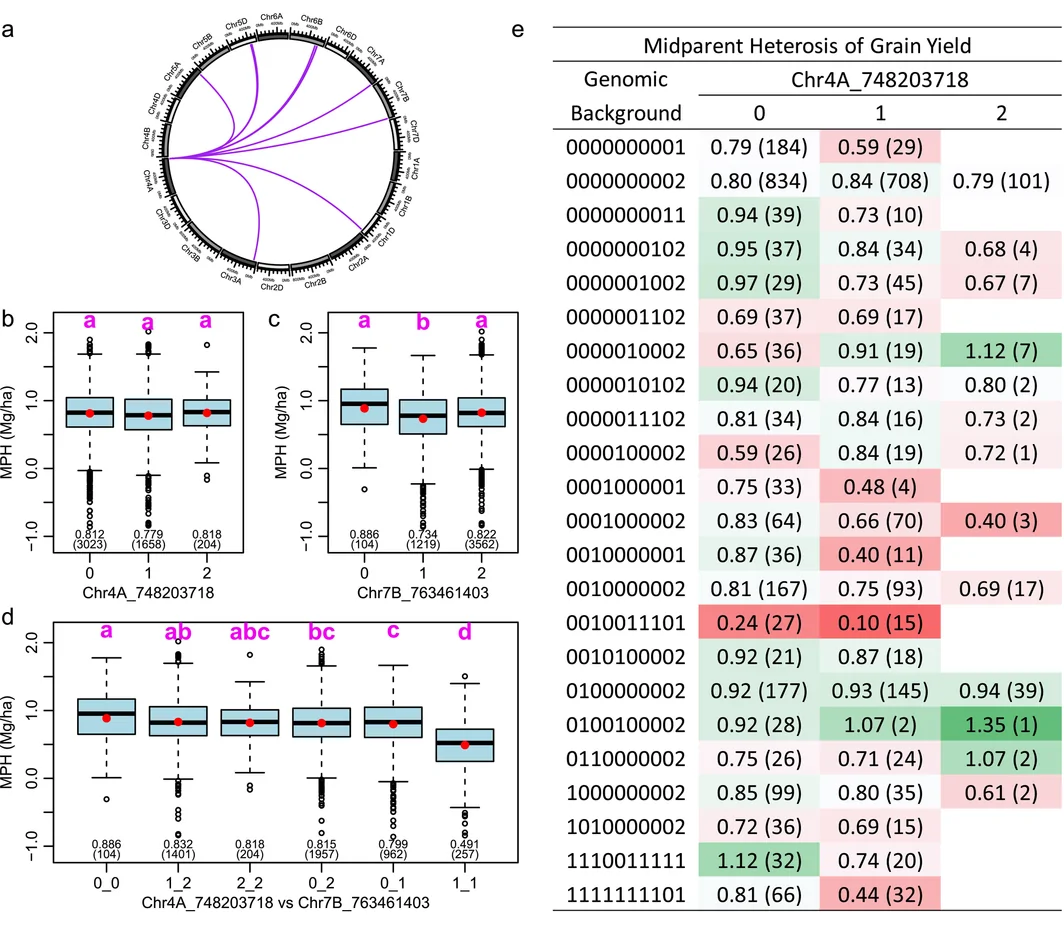
The interaction between Chr4A_748203718 and the rest of genomic background for MPH of grain yield. (a) 10 SNPs with the most significant epistatic effect with Chr4A_748203718 were selected. Comparison of mid‐parent heterosis with different genotypes of the two most significant SNPs, Chr4A_748203718 (b) and Chr7B_763461403 (c), or the combination of these two loci (d). The two values below the boxplot are the mean of MPH and the number of hybrids. (e) Comparison of mid‐parent heterosis with different combinations between Chr4A_748203718 and the rest of genomic background represented by the 10 SNPs. The values within the parentheses show the number of hybrids.

Another way of exploiting hQTL in hybrid breeding is using them to predict the heterosis of hybrids that have not yet been developed or evaluated. To explore the potential of this approach, we trained different prediction models using one experimental dataset and validated them on the other two, resulting in six prediction scenarios (combinations of training and test sets). Three approaches for predicting MPH were compared: (1) a whole‐genome prediction model (WGP) including the dominance effects of all SNPs and digenic epistatic effects between all pairs of SNPs; (2) an hQTL‐based prediction model (hQTL_GP) incorporating only the heterotic effects of the lead SNPs for all hQTL detected in the training population (see Section 4 for details); and (3) a control model (Non‐hQTL_GP) using the same number of randomly selected SNPs outside the hQTL region as in the hQTL_GP model to serve as a benchmark for assessing the value of hQTL in prediction.

Results showed that the hQTL_GP model produced comparable or, in some cases, superior predictive accuracy to the WGP model (Figure 6), despite that the number of predictors in hQTL‐GP was substantially less in WGP. For instance, across six prediction scenarios for grain yield MPH, the average prediction accuracy was 0.150 for the WGP model and 0.147 for the hQTL_GP model. For plant height MPH, the average accuracy was 0.135 for WGP and 0.136 for hQTL_GP. For heading date MPH, the average accuracies of both models were very low. Nevertheless, the average accuracy of hQTL_GP (0.089) was nearly twice as high as WGP (0.047). On the other hand, the hQTL_GP model outperformed Non‐hQTL_GP in most scenarios, particularly for grain yield heterosis (Figure 6a), emphasizing the unique predictive power of hQTL. The low prediction accuracies for all three traits were not unexpected, as our prediction scenario represents the difficult case of predicting heterosis for hybrids whose parental lines were not evaluated in the training population (Zhao et al. 2015, 2021). Thus, although our findings demonstrated the potential of using hQTL to predict heterosis, further improvements in prediction accuracy are needed before it can be applied effectively in hybrid breeding.

**FIGURE 6:**
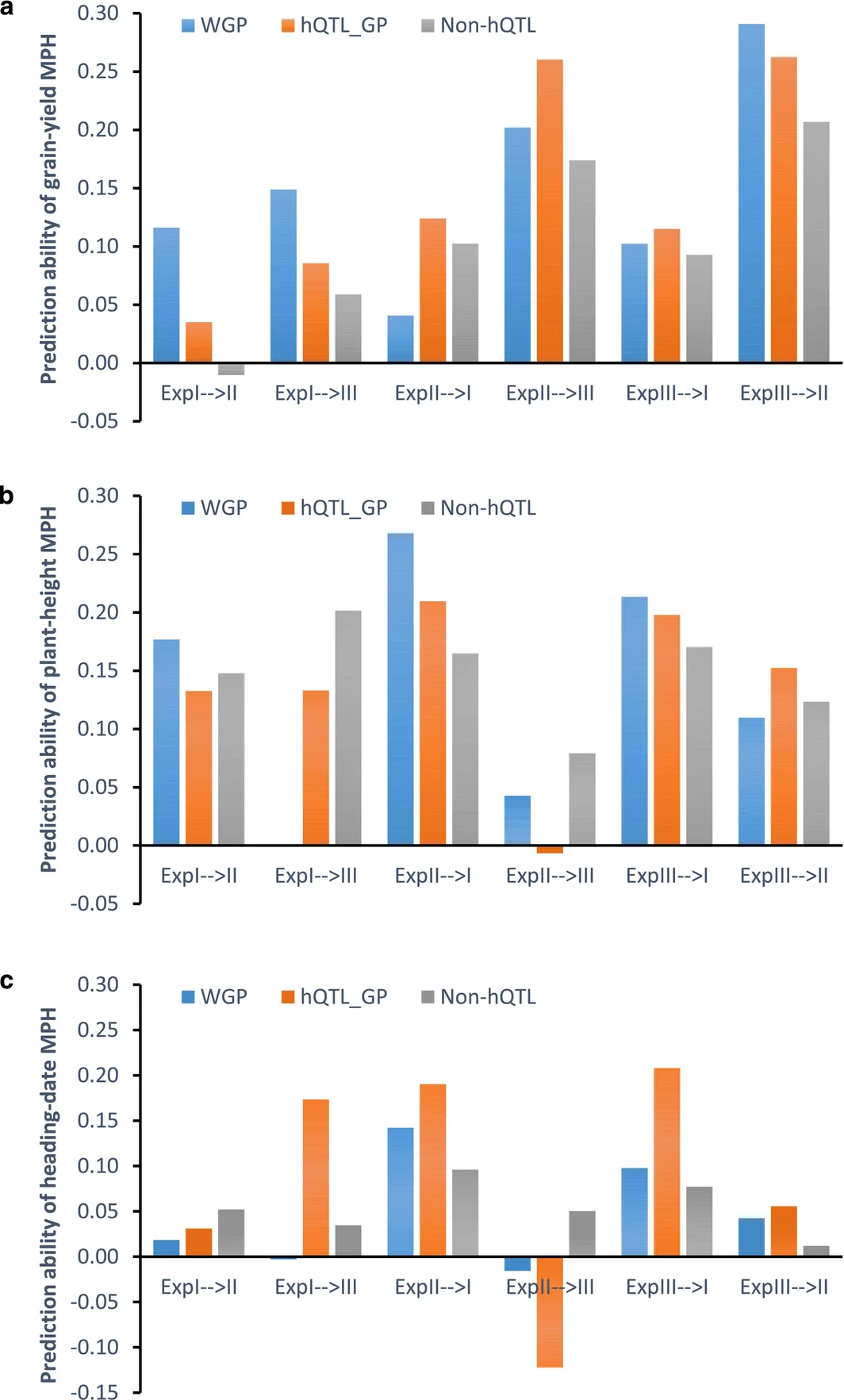
Genomic prediction ability of MPH for grain yield (a), plant height (b), and heading date (c) across experimental series. Six prediction scenarios were designed by combining different training and test sets. In each scenario, prediction models were trained on one experimental dataset and validated on the other two. For instance, Exp I➔ II means prediction model trained on Exp I and validated on Exp II.

## 3 Discussion

By analyses of the specific *Piko* × *Hermann* hybrid and those of a large and genetically diverse hybrid wheat population, we obtained a comprehensive view of the genetic architecture underlying wheat heterosis. Collectively, our analyses revealed that wheat heterosis is predominantly driven by epistasis. Although numerous genomic regions contributed to heterosis, several epistatic hubs exhibiting exceptionally large numbers of interactions were identified, most notably a region on chromosome 4A encompassing a large emmer wheat introgression. These findings contrast with earlier large‐scale studies in other crops such as rice (Gu et al. 2023; Huang et al. 2016), which suggested that loci with dominance effects contribute more substantially to phenotypic variance than overdominance loci, and with studies in maize (Liu et al. 2020) showing that heterosis is mainly governed by numerous loci displaying complete‐to‐incomplete dominance together with a smaller proportion showing overdominance. However, a recent analysis of the 18 K‐rice population (Wei et al. 2024) also identified extensive epistatic QTL pairs and further constructed a genetic interaction network highlighting the central importance of 19 gene hubs. Although grain yield represents the most direct measure of agronomic hybrid performance, the genetic basis of heterosis may differ among agronomic traits. Traits such as plant height and heading date are influenced by several major genes (Beales et al. 2007; Peng et al. 1999; Yan et al. 2003), which may lead to genetic architectures distinct from those underlying grain yield heterosis. Summarizing, the genetic architecture underlying heterosis is highly population‐ and trait‐specific, but also that the contribution of epistasis to heterosis becomes increasingly evident as population size expands and more powerful analytical approaches become available (Li et al. 2025; Wei et al. 2024).

The transition from a hybrid population, which represents parents with broad genetic diversity, to a specific hybrid cross revealed a substantially less complex hQTL landscape. In the *Piko* × *Hermann* biparental population, a single genomic region located in chromosome 4B was the only hQTL detected. An extensive candidate gene prioritization effort narrowed the candidate interval to six genes. While it is tempting to speculate that *Rht‐B1*, a key gene of the Green Revolution, is the causal gene underlying the hQTL, further validation is required. Nevertheless, the relatively small size of the biparental population may limit the resolution of QTL mapping and the precision of defining candidate intervals, emphasizing the need for cautious interpretation of these results.

Benefiting from the high‐density whole‐genome markers of the genetically diverse hybrid wheat population, we were able to refine candidate gene prioritization and consistently identify *TraesCS7B03G1341000* (*TaGNAC1‐7B*) as a strong candidate underlying grain yield heterosis. Publicly available gene expression data from the Wheat Expression Browser (https://www.wheat‐expression.com/) based on the ‘Developmental time‐course of Chinese Spring’ dataset (Choulet et al. 2014) showed that this gene is specifically expressed in grain tissues, with transcript abundance reaching 15.5 transcripts per million (TPM), whereas virtually no expression was detected in root, stem, leaf, or spike tissues. Moreover, its homologous gene, wheat *NAC‐A18* (*TraesCS7A03G1388600*), has been reported to regulate grain starch and storage protein synthesis and to affect grain weight (Wang, Liu, et al. 2023). Collectively, these findings provide strong support for *TraesCS7B03G1341000* as a candidate gene contributing to grain yield heterosis.

Although individual candidate genes may contribute to heterosis, the effects captured by an hQTL are more likely to reflect the collective contribution of multiple linked functional loci and their interaction. From a genetic perspective, the chromosome 4A introgression region (from the *Integrated_GY_hQTL121*) appears to contribute to wheat heterosis through the combined effects of multiple functional loci and their interactions with loci distributed across the genome, rather than through a single major‐effect gene. However, elucidating the underlying biological mechanisms remains challenging because this region represents a large alien introgression containing numerous genes and potentially complex structural variations. These characteristics complicate the identification of causal variants and the functional dissection of individual loci within the introgressed segment. Future studies integrating wheat pan‐genome resources, high‐resolution structural variation analyses, and functional validation will be essential to further resolve how such introgressed regions reshape genetic interactions and contribute to heterosis.

Although MPH was selected as the unified heterosis measure in this study, better‐parent heterosis (BPH) represents another important aspect of hybrid performance with direct relevance to breeding applications. Future dissection of the genetic architecture underlying BPH may provide complementary insights into the genetic mechanisms underlying different manifestations of heterosis.

Dissecting the genetic basis of heterosis is ultimately important because it can provide valuable guidance for practical hybrid breeding. The development of divergent heterotic groups has long been a widely adopted strategy in hybrid breeding (Haberer and Mayer 2022; Melchinger and Gumber 1998). Theoretically, in the absence of epistasis, divergent heterotic groups are expected to increase additive genetic variance relative to dominance variance (Reif et al. 2007), thus simplifying the selection of superior hybrids. However, our results clearly demonstrate that the absence of epistasis does not apply to wheat. This presents a major challenge for breeding because favourable epistatic effects contributing to heterosis are inherently dependent on specific allele combinations and are therefore difficult to efficiently capture and fix in practical hybrid breeding programs. One possible strategy may involve integrating hQTL information into genome‐wide prediction frameworks. In the present study, independent validation experiments showed that prediction models based solely on identified hQTL achieved prediction accuracies comparable to, or even exceeding, those of genome‐wide prediction models. However, the current prediction accuracy remains insufficient for predicting the heterosis of hybrids whose parental lines are not evaluated in the training population, which hampers reliable assembly of superior haplotype combinations. Another potential avenue may involve exploiting epistatic hubs together with their associated interaction nodes to optimize parental selection in hybrid breeding. For example, breeding schemes incorporating the estimated effects of epistatic interaction networks, potentially combined with optimization approaches such as simulated annealing algorithms (Zhao et al. 2015), may facilitate the identification of parental combinations with favourable multi‐locus interactions. Notably, such algorithms have already shown potential for identifying heterotic patterns in hybrid breeding of autogamous crops (Zhao et al. 2015), suggesting that optimization approaches based on simulated annealing or other similar algorithms and incorporating epistatic interaction networks into parental selection may provide a promising avenue for accelerating hybrid wheat breeding in the future.

## 4 Materials and Methods

### 4.1 QTL Mapping for Heterosis in the Biparental Population of *Piko* × *Hermann*

Two Central European winter wheat cultivars, *Piko* and *Hermann*, were selected to generate a double haploid (DH) population comprising 89 DH lines. This DH population served as the founder population for constructing both TTC and IMF_2_ populations (Figure 1b). Three testcross (TC) populations were produced by crossing each of the 89 DH lines with one of the three testers: *Piko*, *Hermann*, and their F_1_ hybrid (*Piko* × *Hermann*). These populations were denoted as TCP, TCH, and TCF, respectively. In parallel, the IMF_2_ population was generated using single round‐robin crosses among the 89 DH lines, resulting in 80 single‐cross hybrids (Figure 1b). In total, 439 genotypes comprising *Piko*, *Hermann*, *Piko* × *Hermann*, 89 individuals in each of four populations (DH, TCP, TCH, TCF), and 80 in IMF_2_ were included in this study. All genotypes were evaluated in field trials using an alpha‐lattice design with two replications. Multi‐environment field trials were conducted at four locations (Gatersleben, Biendorf, Schackstedt, and Mollochshöhe) in Germany from 2021 to 2023. Phenotypic data were collected for heading date (days from January 1st) in 9 environments (year‐by‐location combinations), plant height (cm) in 11 environments, and grain yield (Mg/ha) in 7 environments (Table S1). Phenotypic evaluations were conducted under field conditions. Grain yield was measured in plots ranging from 4.5 to 8.7 m^2^, with a sowing density of 300 seeds m^−2^. All details of phenotypic and genotypic data are provided in Supporting Information.

The exceptional feature of the TTC design is its applicability for the identification of QTL contributing to heterosis (Melchinger et al. 2008). For a single hybrid whose parents are inbred lines, the net contribution of a locus to its MPH was termed augmented dominance effect ($d_{i}^{*}$), defined as $d_{i}^{*}=d_{i}-\frac{1}{2}\sum_{j\neq i}{\mathrm{aa}}_{\mathrm{ij}}$, where $d_{i}$ is the dominance effect of the *i*th marker and ${\mathrm{aa}}_{\mathrm{ij}}$ is the additive‐by‐additive epistatic effect between the *i*th and the *j*th marker (Melchinger et al. 2007). Remarkably, the augmented dominance effect can be directly tested in a one‐dimensional scan by using classic QTL mapping approach (Melchinger et al. 2007), which we briefly recall as follows.

First of all, for each trait, phenotypic data analysis was performed by combining the data from all environments and we obtained the best linear unbiased estimators (BLUEs) of the genetic values for all progenies in the populations DH, TCP, TCH, TCF, and IMF_2_. Details on the phenotypic data analysis were provided in Supporting Information.

We denote the BLUEs of the individuals in the TCP, TCH, and TCF population by $H_{1k},H_{2k},$ and $H_{3k}\;\left(k=1,2,\ldots ,n\right)$, respectively, where n is the size of the DH population (n=89 in this study). Then, a derived phenotype was calculated as $Z_{2k}=H_{1k}-H_{2k}$. To test the augmented dominance effect for each marker, the following two linear models were compared:

(1) $$Z_{2}=1_{n}\mu +\sum_{q=1}^{c}X_{q}d_{q}^{*}+\varepsilon$$

(2) $$\begin{array}{c} Z_{2}=1_{n}\mu +X_{i}d_{i}^{*}+\sum_{q=1}^{c}X_{q}d_{q}^{*}+\varepsilon \end{array}$$

where $Z_{2}$ is the vector consisting of $Z_{2k}$ $\left(k=1,2,\ldots ,89\right)$, $1_{n}$ is an *n*‐dimensional vector of ones, μ is the common intercept, $d_{q}^{*}$ is the augmented dominance effect of the q‐th marker as a cofactor, $d_{i}^{*}$ is the augmented dominance effect of the marker being tested, $X_{q}$ and $X_{i}$ are the corresponding coding vectors, ε is the residual vector. In the models, all unknown parameters were treated as fixed effects except for the residuals. The marker cofactors were preselected by using a stepwise linear regression model based on the Akaike's information criterion (AIC; Sakamoto et al. 1986), and c is the number of cofactors. It is important to note that the marker coding vectors correspond to the genotypes in the DH population (instead of those in TCP, TCH or TCF), and cofactors within 1 Mbp window flanking the marker being tested were excluded. An *F*‐test was used to assess the significance of $d_{i}^{*}$ (Reif et al. 2010).

While the above approach directly tests the augmented dominance effect $d_{i}^{*}$, it is also interesting to know whether its component effects (i.e., the dominance effect $d_{i}$ and the epistatic effects ${\mathrm{aa}}_{\mathrm{ij}}$ for all j≠i) are significant or not. The TTC design enables a two‐dimensional scan for the additive‐by‐additive epistatic effects by using the following linear models (Melchinger et al. 2008):

(3) $$\begin{array}{c} H_{3}=1_{n}\mu +X_{i}a_{i}+X_{j}a_{j}+\sum_{q=1}^{c}X_{q}a_{q}+\varepsilon \end{array}$$

(4) $$\begin{array}{c} H_{3}=1_{n}\mu +X_{i}a_{i}+X_{j}a_{j}+\left(X_{i}\circ X_{j}\right){\mathrm{aa}}_{\mathrm{ij}}+\sum_{q=1}^{c}X_{q}a_{q}+\varepsilon \end{array}$$

where $H_{3}$ is the vector consisting of $H_{3k}\;\left(k=1,2,\ldots ,89\right)$, $a_{q}$ is the additive effect of the i‐th marker as cofactor, $a_{i}$ and $a_{j}$ are the additive effects of the *i*‐th and the *j*‐th marker, ${\mathrm{aa}}_{\mathrm{ij}}$ is the additive‐by‐additive effect between the two markers, $X_{q}$, $X_{i}$ and $X_{j}$ are the corresponding marker coding vectors, and ε is the residual vector. An *F*‐test comparing the reduced model (3) and the full model (4) was used to evaluate the significance of ${\mathrm{aa}}_{\mathrm{ij}}$.

Unfortunately, it is difficult to disentangle the contribution of the dominance effect alone with the TTC design (Melchinger et al. 2008). Thus, we complemented the above analysis with a QTL mapping for dominance effects in the IMF_2_ population by using the same QTL mapping approach as for the augmented dominance effects. Namely, in models (1) and (2), the vectors $Z_{2}$, $X_{q}$, $X_{i}$, $d_{q}^{*}$ and $d_{i}^{*}$ were replaced by y, $M_{q}$, $M_{i}$, $d_{q}$ and $d_{i}$, respectively, where y is the vector of BLUEs for the individuals in the IMF_2_ population, $d_{q}$ and $d_{i}$ are the dominance effects of the *q*th and the *i*th marker, $M_{i}$ and $M_{q}$ are the corresponding marker coding vectors. Here, the marker coding refers to the genotype of the individuals in the IMF_2_ population.

In all cases, the F_2_‐metric was used for marker coding (Zeng et al. 2005), and the significance threshold was determined to be *p* < 0.05 after correction based on 1000 permutation tests (Churchill and Doerge 1994). Cofactor selection and QTL mapping were conducted using statistical software R (v4.1.0, R Development Core Team (2021)).

### 4.2 Heterotic QTL Identification and Testing of Its Genetic Component Effects in the Diverse Hybrid Population

A total of 597 wheat elite parental lines and 5243 F_1_ hybrid progenies from three large hybrid wheat populations (Experimental series I, II and III; Li et al. 2025; Zhao et al. 2021) were utilized for this study (see Supporting Information for details about materials and whole‐genome sequencing (WGS) data). MPH values were calculated based on BLUEs estimated across all environments (defined as combinations of location and year).

To overcome the computational bottleneck caused by high‐density markers, GWAS for detecting hQTL was conducted using the hQTL‐ODS model ((Li et al. 2025), https://github.com/Ligl0226/hQTL‐ODS/). Briefly, for each marker, the following two LMMs are compared.

(5) $$\begin{array}{c} y_{\mathrm{MPH}}=g_{D}+g_{\mathrm{AA}}+g_{\mathrm{AD}}+g_{\mathrm{DD}}+\varepsilon_{\mathrm{MPH}}, \end{array}$$

(6) $$\begin{array}{c} y_{\mathrm{MPH}}=h_{i}+g_{D}+g_{\mathrm{AA}}+g_{\mathrm{AD}}+g_{\mathrm{DD}}+\varepsilon_{\mathrm{MPH}}, \end{array}$$

where ***y***_*MPH*_ is the vector of MPH values for all hybrids and ε is the residual. Model (5) is the null model containing multiple genetic background effects $g_{D}\sim N\left(0,K_{D}\sigma_{D}^{2}\right)$, $g_{\mathrm{AA}}\sim N\left(0,K_{\mathrm{AA}}\sigma_{\mathrm{AA}}^{2}\right)$, $g_{\mathrm{AD}}\sim N\left(0,K_{\mathrm{AD}}\sigma_{\mathrm{AD}}^{2}\right)$, and $g_{\mathrm{DD}}\sim N\left(0,K_{\mathrm{DD}}\sigma_{\mathrm{DD}}^{2}\right)$, where $K_{*}$ (* denotes D, AA, AD, or DD) is the genomic kinship matrix that accounts for a specific type of genetic background effects (depending on *) and $\sigma_{*}^{2}$ is the corresponding variance component. Note that$\;\varepsilon_{\mathrm{MPH}}\sim N\left(0,{\mathrm{TT}}^{\prime }\sigma_{\varepsilon }^{2}\right)$, where T is the matrix of linear transformation from the (*n* + *r*)‐dimensional vector of original trait values to the *n*‐dimensional vector of MPH values (*n* is the number of hybrids and *r* is the number of parents). The alternative model (6) includes the heterotic effect (***h***_*i*_) of the tested marker in addition to all effects in the null model. The heterotic effect ***h***_*i*_ is defined as a complex linear combination of (4p‐3) effects, that is, the dominance effect $d_{i}$ and the epistatic effects ${\mathrm{aa}}_{\mathrm{ij}}$, ${\mathrm{ad}}_{\mathrm{ij}}$, ${\mathrm{da}}_{\mathrm{ij}}$, ${\mathrm{dd}}_{\mathrm{ij}}$ (j=1,…,p and j≠i, where p is the number of markers) (Jiang et al. 2017; Li et al. 2025). And, $h_{i}$ is treated as random effect following a multi‐variate normal distribution, $h_{i}\sim N\left(0,H_{i}\sigma_{i}^{2}\right)$, where covariance matrix $H_{i}$is constructed based on the definition of $h_{i}$ and $\sigma_{i}^{2}$ is the corresponding variance component (Li et al. 2025). The likelihood ratio test is used to assess the significance of $h_{i}$.

When a marker has significant heterotic effect ***h***_*i*_, to further clarify the contribution of its individual component effects, we performed significance tests for the dominance effects *d*_*i*_ and all four types of epistatic effects, namely ${\mathrm{aa}}_{\mathrm{ij}}$, ${\mathrm{ad}}_{\mathrm{ij}}$, ${\mathrm{da}}_{\mathrm{ij}}$ and ${\mathrm{dd}}_{\mathrm{ij}}$ for all *j* ≠ *i*, 1 ≤ *j* ≤ *p*. The following model was used for testing these effects.

(7) $$\begin{array}{c} y_{\mathrm{MPH}}=\mathrm{M\alpha}+g_{D}+g_{\mathrm{AA}}+g_{\mathrm{AD}}+g_{\mathrm{DD}}+\varepsilon_{\mathrm{MPH}} \end{array}$$

where α is a vector consisting of the individual genetic effects being tested and necessary covariates (Table S36), M is the corresponding design matrix, and other notations are the same as in model (5,6). α is treated as a vector of fixed variables and the significance of the tested genetic effect is assessed by the Wald test. *p* values were corrected for multiple testing with the Bonferroni method and the significance level was determined as 0.05. To reduce the computational burden of testing large number of pairwise interactions, tagging SNPs were selected to represent the hQTL. Significant SNPs were considered independent if they were separated by > 1 Mb and had a linkage disequilibrium (LD) value of *r* ^2^ < 0.8; within each group, the SNP with the lowest *p*‐value was selected as a tagging SNP.

### 4.3 The Emmer Wheat Introgression Survey on the Distal End of Chromosome 4A

A previously reported introgression from emmer wheat (Schulthess et al. 2022; Walkowiak et al. 2020), located on chromosome 4A (from 732 979 379 to 754 227 511 bps based on CSv2.1), overlaps with a key heterotic QTL identified in our study (*Integrated_GY_hQTL121*). However, the presence/absence information for this alien introgression was available only for the parental lines in experimental series I (Schulthess et al. 2022). To extend this information across the entire integrated population, we performed principal coordinate analysis (PCoA) using SNPs mapping to the introgression region. By comparing the pattern of clustering in the integrated population with that of experimental series I (Figure S9), we inferred the presence or absence of the introgression for each parental line in the integrated population (Table S37). Notably, the significant SNP (Chr4A_748203718) within hQTL *Integrated_GY_hQTL121* was perfectly correlated with the presence/absence status of the introgression, thereby emerging as a promising diagnostic marker for this alien introgression in subsequent analyses.

### 4.4 Genome‐Wide Prediction of MPH Across Different Experimental Series

To validate the capability of the hQTL to characterize the genetic architecture of MPH and to explore the potential of using hQTL for predicting MPH, we evaluated three genomic prediction models in six different scenarios. In each scenario, one experimental series served as training set and another was treated as the test set. Since there are only a limited number of common parental lines between different experimental series, the prediction scenarios represent very close to the so‐called T0 case (Zhao et al. 2021), which is the most challenging scenario for predicting the performance of hybrids. For this part of the study, we used 644 876 high‐quality SNPs shared among the genotypes across the three experimental series (Figure S3). In the following, we briefly describe the three genomic prediction models:

The first model is a whole‐genome prediction (WGP) model incorporating both dominance and digenic epistatic effects of all markers across the genome, which is the same as model (5). This model served as the baseline and it was implemented in the same way as in (Jiang et al. 2017).

In the second model, denoted as hQTL_GP, we only considered the heterotic effects of the lead SNPs in all hQTL identified in the training population. The model is of the following form:

(8) $$\begin{array}{c} y_{\mathrm{MPH}}=\sum_{i\in Q}h_{i}+\varepsilon_{\mathrm{MPH}}, \end{array}$$

where ***y***_*MPH*_ is the vector of observed MPH values of the trait, ***h***_*i*_ is the vector of heterotic effects for the *i*th marker, *Q* is the set of lead SNPs (one for each hQTL), and ***ε*** is the residuals. More precisely, ***h***_*i*_ is defined as follows (Li et al. 2025):

(9) $$\begin{array}{c} h_{i}=T\left[l_{i}d_{i}+\frac{1}{2}\sum_{\begin{array}{c} j=1\\ j\neq i \end{array}}^{p}\left(\left(m_{i}\circ m_{j}\right){\mathrm{aa}}_{\mathrm{ij}}+\left(m_{i}\circ l_{j}\right){\mathrm{ad}}_{\mathrm{ij}}+\left(l_{i}\circ m_{j}\right){\mathrm{ad}}_{\mathrm{ji}}+\left(l_{i}\circ l_{j}\right){\mathrm{dd}}_{\mathrm{ij}}\right)\right], \end{array}$$

where ***T*** is the same as defined in Equations (5) and (6), p is the number of all markers, ***m***_*i*_ and ***m***_*j*_ are the *i*th and the *j*th columns of the additive marker coding matrix ***M***_*A*_, ***l***_*i*_ and ***l***_*j*_ are the *i*th and the *j*th columns of the dominance marker coding matrix ***M***_*D*_, and ‘∘’ denotes entry‐wise product of two vectors.

Since the definition of ***h***_*i*_ is complex, to efficiently implement the model, we assumed that all component effects ($d_{i}$, ${\mathrm{aa}}_{\mathrm{ij}}$, ${\mathrm{ad}}_{\mathrm{ij}}$, ${\mathrm{ad}}_{\mathrm{ji}}$ and ${\mathrm{dd}}_{\mathrm{ij}}$) are independent random variables, each following a normal distribution with zero mean and a common variance $\sigma_{h}^{2}$. Then, we have $h_{i}∼N\left(0,H_{i}\sigma_{h}^{2}\right)$, where $H_{i}=Z_{i}Z_{i}^{\prime }$, $Z_{i}$ is the $n\times \left(4p-3\right)$ matrix whose columns comprise $Tl_{i}$, $\frac{1}{2}T\left(m_{i}\circ m_{j}\right)$, $\frac{1}{2}T\left(m_{i}\circ l_{j}\right)$, $\frac{1}{2}T\left(l_{i}\circ m_{j}\right)$ and $\frac{1}{2}T\left(l_{i}\circ l_{j}\right)$, for all 1≤j≤p and j≠i. Thus, the model (9) can be rewritten as follows:

(10) $$\begin{array}{c} y_{\mathrm{MPH}}=h+\varepsilon_{\mathrm{MPH}}, \end{array}$$

where $h=\sum_{i\in Q}h_{i}∼N\left(0,H\sigma_{h}^{2}\right)$, $H=\sum_{i\in Q}H_{i}$. Once the covariance matrix H is calculated, this model is no more complex than a usual genomic best linear unbiased prediction (GBLUP) model, except that $\varepsilon_{\mathrm{MPH}}\sim N\left(0,{\mathrm{TT}}^{\prime }\sigma_{\varepsilon }^{2}\right)$ instead of $N\left(0,I\sigma_{\varepsilon }^{2}\right)$. The way of efficiently calculating H and dealing with non‐independent residuals was the same as in (Li et al. 2025).

The third model, termed Non‐hQTL_GP, followed the same structure as hQTL_GP. The only difference is that the lead SNPs of hQTL were replaced by an equal number of randomly selected SNPs not belonging to any hQTL. This model served as a benchmark to assess the added value of hQTL in prediction.

## Author Contributions

J.C.R. and Y.J. designed and supervised the project. G.L. performed phenotype processing, hQTL‐ODS analysis, GWAS analysis, QTL mapping, and genomic prediction with the support of all other co‐authors. J.C. and M.H. performed the SNP calling. G.L., J.C.R. and Y.J. wrote the manuscript.

## Funding

The work was supported by the German Research Foundation (DFG) within the Project ‘Developing a statistical genomics toolbox to decipher the genetic architecture of heterosis using whole‐genome sequencing data’ (grant number: 540803247). The field trial data and analyses of the *Piko* × *Hermann* cross was supported in the frame of the Hyflor project financed by the Federal Ministry of Food and Agriculture (grant nr. FKZ2818401A18). Jochen Reif activities were supported in the frame of the TWIN project funded by the German Federal Ministry of Research, Technology and Space (grant no. FKZ031B1557A). FAIRification of the data was supported in the frame of the NFDI consortium FAIRagro (www.fairagro.net). We gratefully acknowledge the financial support of DFG (grant number 501899475).

## Conflicts of Interest

The authors declare no conflicts of interest.

## Supporting information


**Figure S1:** Grain yield performance of the *Piko* × *Hermann* hybrid and its parental lines across environments.
**Figure S2:** Genetic architecture of midparent heterosis for heading date (days) in the hybrid *Piko* × *Hermann*. (a) The genome wide scan of hQTL within the TTC population. (b) A genome‐wide scan of the dominance was conducted within IMF_2_ population.
**Figure S3:** Schematic illustration of whole‐genome re‐sequencing data curation, GWAS mapping analysis and whole genome‐wide prediction of MPH.
**Figure S4:** Manhattan and QQ plot for heterotic effects and dominance effects in the integrated population. (a, c, e) Plot for heterotic effect of grain yield, heading date, and plant height. (b, d, f) Plot for dominance effect of grain yield, heading date, and plant height.
**Figure S5:** Heatmap of linkage disequilibrium (LD) between all SNPs from the distal end of chromosome 4A, spanning the region from 602.37 Mb to 754.16 Mb.
**Figure S6:** The Manhattan plot of chromosome 4A for heterotic effect within integrated population (a) and subpopulation classified by SNP Chr4A_748203718, 0 means without introgression (b) and 2 means with introgression (c).
**Figure S7:** Manhattan plot of epistatic interaction between significant SNPs (Chr4A_748203718 (a) and Chr7B_763461403 (b)) with the remaining genomic background for grain yield.
**Figure S8:** Schematic overview of the data‐driven approach for candidate gene prioritization by retrieving all SNPs within hQTL regions and integrating association analyses from individual environments within each experimental series.
**Figure S9:** Principal coordinate analysis (PCoA) plots of the first two components for parental lines from experimental series I (a) and Integrated population (b) based on the Rogers' distance matrix calculated using SNPs located in the distal end of chromosome 4A introgression genomic region (from Chr4A: 732 979 379 to 754 227 511).
**Appendix S1:** Supplementary Notes.

**Table S1:** Performance of *Piko* × *Hermann* and variance analysis in the biparental population.
**Table S2:** Analysis of variance for the contrast Z3 in TTC design.
**Table S3:** Significant additive‐by‐additive epistatic effects identified by two‐dimensional genome‐wide scanning using the performance of crosses between doubled haploid (DH) lines and the *Piko* × *Hermann* hybrid in the TTC design.
**Table S4:** Candidate wheat genes carrying putatively functional variants within the bin interval of the significant grain yield heterosis marker TG0010b, together with their corresponding *Arabidopsis thaliana* homologues and functional annotations.
**Table S5:** Overview of the phenotype data within the three experimental series and integrated population.
**Table S6:** hQTL identified in integrated population for grain yield.
**Table S7:** dQTL identified in integrated population for grain yield.
**Table S8:** hQTL identified in integrated population for heading date.
**Table S9:** dQTL identified in integrated population for heading date.
**Table S10:** hQTL identified in integrated population for plant height.
**Table S11:** dQTL identified in integrated population for plant height.
**Table S12:** hQTL identified in experimental series I for grain yield.
**Table S13:** dQTL identified in experimental series I for grain yield.
**Table S14:** hQTL identified in experimental series I for heading date.
**Table S15:** dQTL identified in experimental series I for heading date.
**Table S16:** hQTL identified in experimental series I for plant height.
**Table S17:** dQTL identified in experimental series I for plant height.
**Table S18:** hQTL identified in experimental series II for grain yield.
**Table S19:** hQTL identified in experimental series II for heading date.
**Table S20:** hQTL identified in experimental series II for plant height.
**Table S21:** hQTL identified in experimental series III for grain yield.
**Table S22:** hQTL identified in experimental series III for heading date.
**Table S23:** dQTL identified in experimental series III for heading date.
**Table S24:** hQTL identified in experimental series III for plant height.
**Table S25:** dQTL identified in experimental series III for plant height.
**Table S26:** Summary of hQTL identified for three traits across four panels.
**Table S27:** Summary of dQTL identified for three traits across four panels.
**Table S28:** Overlap check of all hQTL and dQTL identified in the integrated population.
**Table S29:** Comprehensive annotation of the 15 grain yield hQTL‐CGR regions, including hQTL detected using BLUEs across environments within each of the three experimental series, gene annotations, and SNP variant information.
**Table S30:** Comprehensive annotation of the 15 grain yield hQTL‐CGR regions, including hQTL detected within each of the 35 environments, gene annotations, and SNP variant information.
**Table S31:** Comprehensive annotation of the 16 heading date hQTL‐CGR regions, including hQTL detected using BLUEs across environments within each of the three experimental series, gene annotations, and SNP variant information.
**Table S32:** Comprehensive annotation of the 16 heading date hQTL‐CGR regions, including hQTL detected within each of the 48 environments, gene annotations, and SNP variant information.
**Table S33:** Comprehensive annotation of the 28 plant height hQTL‐CGR regions, including hQTL detected using BLUEs across environments within each of the three experimental series, gene annotations, and SNP variant information.
**Table S34:** Comprehensive annotation of the 28 plant height hQTL‐CGR regions, including hQTL detected within each of the 54 environments, gene annotations, and SNP variant information.
**Table S35:** ANOVA of MPH using SNP Chr4A_748203718 and its 10 most significant epistatic partner SNPs.
**Table S36:** The component effects of a hQTL to be screen in model (7).
**Table S37:** Absence/presence of alien introgressions (end of chromosome 4A) for each genotype in the integrated population.

## Data Availability

Raw whole‐genome sequencing reads of all parental lines in the integrated population are available on European Nucleotide Archive (https://www.ebi.ac.uk/ena/) under the Project IDs: PRJEB48738 (Schulthess et al. 2022) and PRJEB82869 (Li et al. 2025). VCF files for whole WGS data are available from European Variation Archive (https://www.ebi.ac.uk/eva/) under project PRJEB87554 (Li et al. 2025). The phenotypic data of all parents and hybrids are provided in GitHub (https://github.com/Ligl0226/hQTL‐ODS/). Correspondence and requests for materials and data should be addressed to Jochen C. Reif (reif@ipk-gatersleben.de).
