# Supplementary material for: Genetic Dissection of Wheat Heterosis Identifies Candidate Genes in Biparental and Diverse Hybrid Populations
Source: Plant Biotechnol J. 2026 Aug 30;24(11):5848–64. doi: 10.1111/pbi.70748 (PMC13527459; doi:10.1111/pbi.70748)
Supplement: Supplementary file 1 [file PBI-24-5848-s002.zip › pbi70748-sup-0004-FigureS3@Figure S3.pdf]

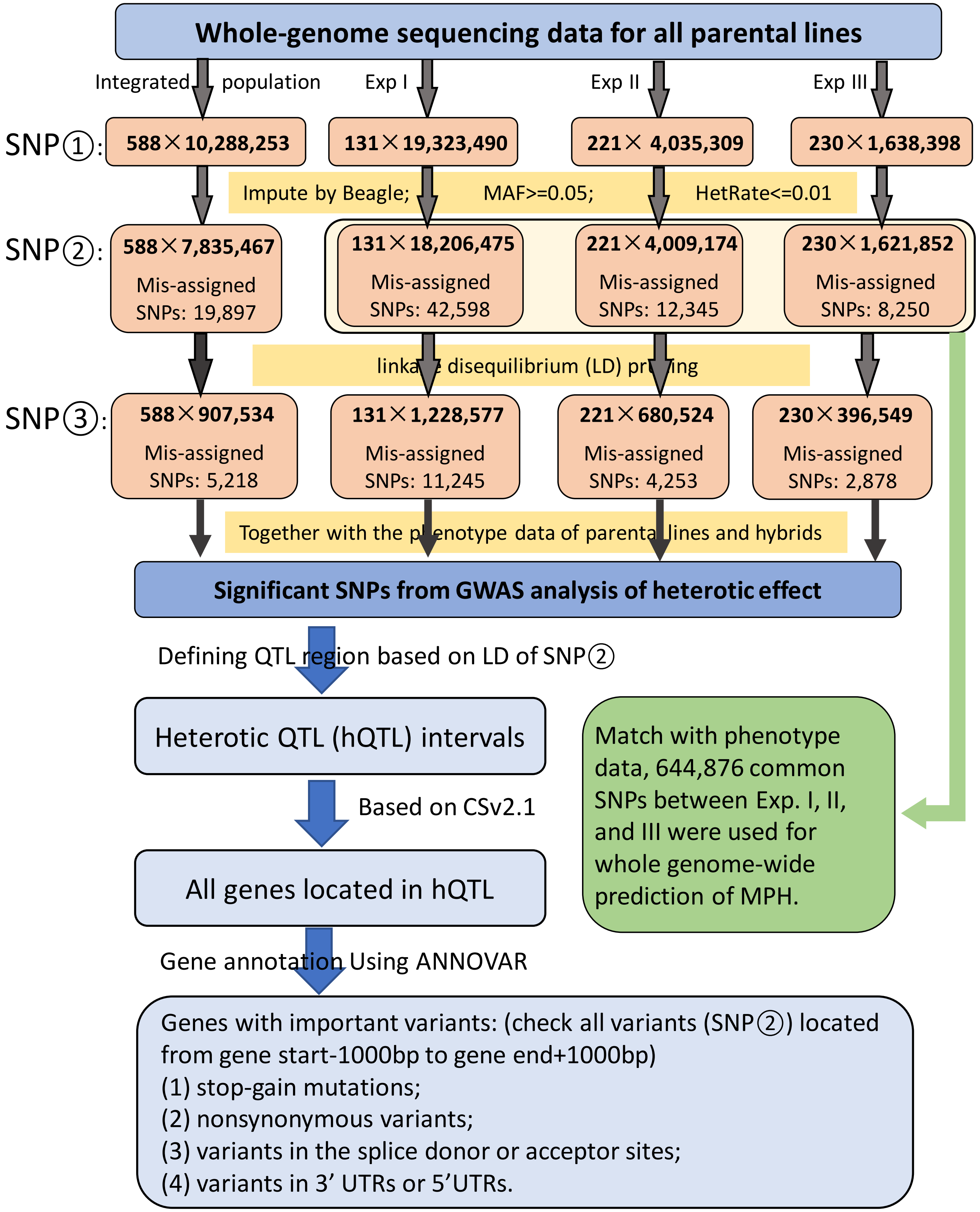
Whole-genome sequencing data for all parental lines
Integrated population
Exp I
Exp II
Exp III
SNP①:
588×10,288,253
131×19,323,490
221× 4,035,309
230×1,638,398
Impute by Beagle; MAF>=0.05; HetRate<=0.01
SNP②:
588×7,835,467
Mis-assigned SNPs: 19,897
131×18,206,475
Mis-assigned SNPs: 42,598
221×4,009,174
Mis-assigned SNPs: 12,345
230×1,621,852
Mis-assigned SNPs: 8,250
linkage disequilibrium (LD) pruning
SNP③:
588×907,534
Mis-assigned SNPs: 5,218
131×1,228,577
Mis-assigned SNPs: 11,245
221×680,524
Mis-assigned SNPs: 4,253
230×396,549
Mis-assigned SNPs: 2,878
Together with the phenotype data of parental lines and hybrids
Significant SNPs from GWAS analysis of heterotic effect
Defining QTL region based on LD of SNP②
Heterotic QTL (hQTL) intervals
Based on CSv2.1
All genes located in hQTL
Match with phenotype data, 644,876 common SNPs between Exp. I, II, and III were used for whole genome-wide prediction of MPH.
Gene annotation Using ANNOVAR
Genes with important variants: (check all variants (SNP②) located from gene start-1000bp to gene end+1000bp)
(1) stop-gain mutations;
(2) nonsynonymous variants;
(3) variants in the splice donor or acceptor sites;
(4) variants in 3' UTRs or 5'UTRs.
