# Supplementary material for: Genetic Dissection of Wheat Heterosis Identifies Candidate Genes in Biparental and Diverse Hybrid Populations
Source: Plant Biotechnol J. 2026 Aug 30;24(11):5848–64. doi: 10.1111/pbi.70748 (PMC13527459; doi:10.1111/pbi.70748)
Supplement: Supplementary file 1 [file PBI-24-5848-s002.zip › pbi70748-sup-0006-FigureS5@Figure S5.pdf]

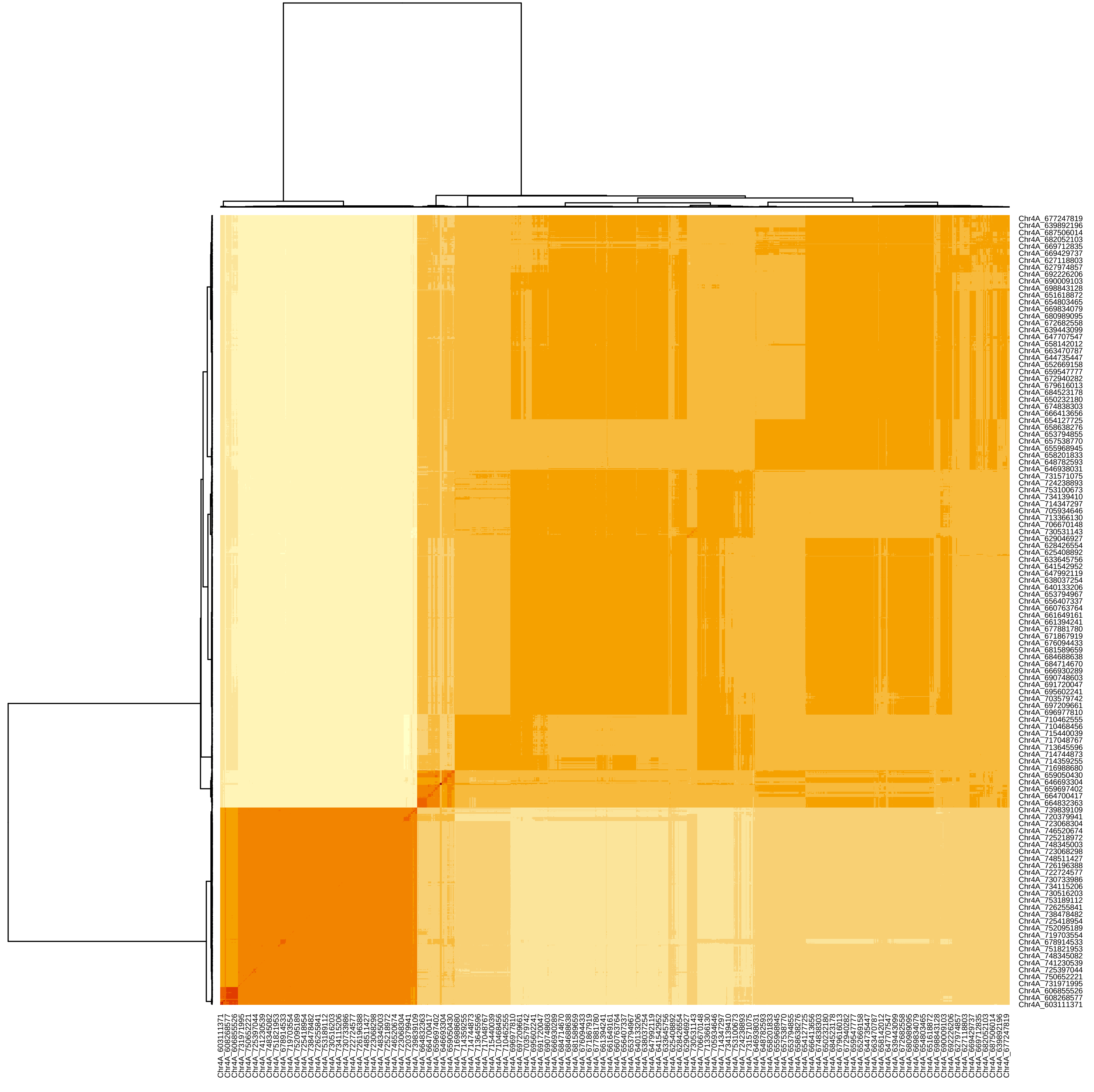
Chr4A_677247819
Chr4A_639892196
Chr4A_687506014
Chr4A_682052103
Chr4A_669712835
Chr4A_669429737
Chr4A_627118803
Chr4A_627974857
Chr4A_692226206
Chr4A_690009103
Chr4A_698843128
Chr4A_651618872
Chr4A_654803465
Chr4A_669834079
Chr4A_680989095
Chr4A_672682558
Chr4A_639443099
Chr4A_647707547
Chr4A_658142012
Chr4A_663470787
Chr4A_644735447
Chr4A_652669158
Chr4A_659547777
Chr4A_679616013
Chr4A_672940282
Chr4A_684523178
Chr4A_650232180
Chr4A_674838303
Chr4A_666413656
Chr4A_654127725
Chr4A_658638276
Chr4A_653794855
Chr4A_657538770
Chr4A_655968945
Chr4A_658201833
Chr4A_648782593
Chr4A_646938031
Chr4A_731571075
Chr4A_724238893
Chr4A_753100673
Chr4A_734139410
Chr4A_714347297
Chr4A_705934646
Chr4A_713366130
Chr4A_706670148
Chr4A_730531143
Chr4A_629046927
Chr4A_628426554
Chr4A_625408892
Chr4A_633645756
Chr4A_641542952
Chr4A_647992119
Chr4A_638037254
Chr4A_640133206
Chr4A_653794967
Chr4A_656407337
Chr4A_660763764
Chr4A_661649161
Chr4A_661394241
Chr4A_677881780
Chr4A_671867919
Chr4A_676094433
Chr4A_681589659
Chr4A_684688638
Chr4A_684714670
Chr4A_666930289
Chr4A_690748603
Chr4A_691720047
Chr4A_695602241
Chr4A_703579742
Chr4A_697209661
Chr4A_696977810
Chr4A_710462555
Chr4A_710468456
Chr4A_715440039
Chr4A_717048767
Chr4A_713645596
Chr4A_714744873
Chr4A_714359255
Chr4A_716988680
Chr4A_659050430
Chr4A_646693304
Chr4A_659697402
Chr4A_664700417
Chr4A_664832363
Chr4A_739839109
Chr4A_720379941
Chr4A_723068304
Chr4A_746520674
Chr4A_725218972
Chr4A_748345003
Chr4A_723068298
Chr4A_748511427
Chr4A_726196388
Chr4A_722724577
Chr4A_730733986
Chr4A_734115206
Chr4A_730516203
Chr4A_753189112
Chr4A_726255841
Chr4A_738478482
Chr4A_725418954
Chr4A_752095189
Chr4A_719703554
Chr4A_678914533
Chr4A_751821953
Chr4A_748345082
Chr4A_741230539
Chr4A_725397044
Chr4A_750652221
Chr4A_731971995
Chr4A_606855526
Chr4A_608268577
Chr4A_603111371
