# Supplementary material for: Genetic Dissection of Wheat Heterosis Identifies Candidate Genes in Biparental and Diverse Hybrid Populations
Source: Plant Biotechnol J. 2026 Aug 30;24(11):5848–64. doi: 10.1111/pbi.70748 (PMC13527459; doi:10.1111/pbi.70748)
Supplement: Supplementary file 1 [file PBI-24-5848-s002.zip › pbi70748-sup-0007-FigureS6@Figure S6.pdf]

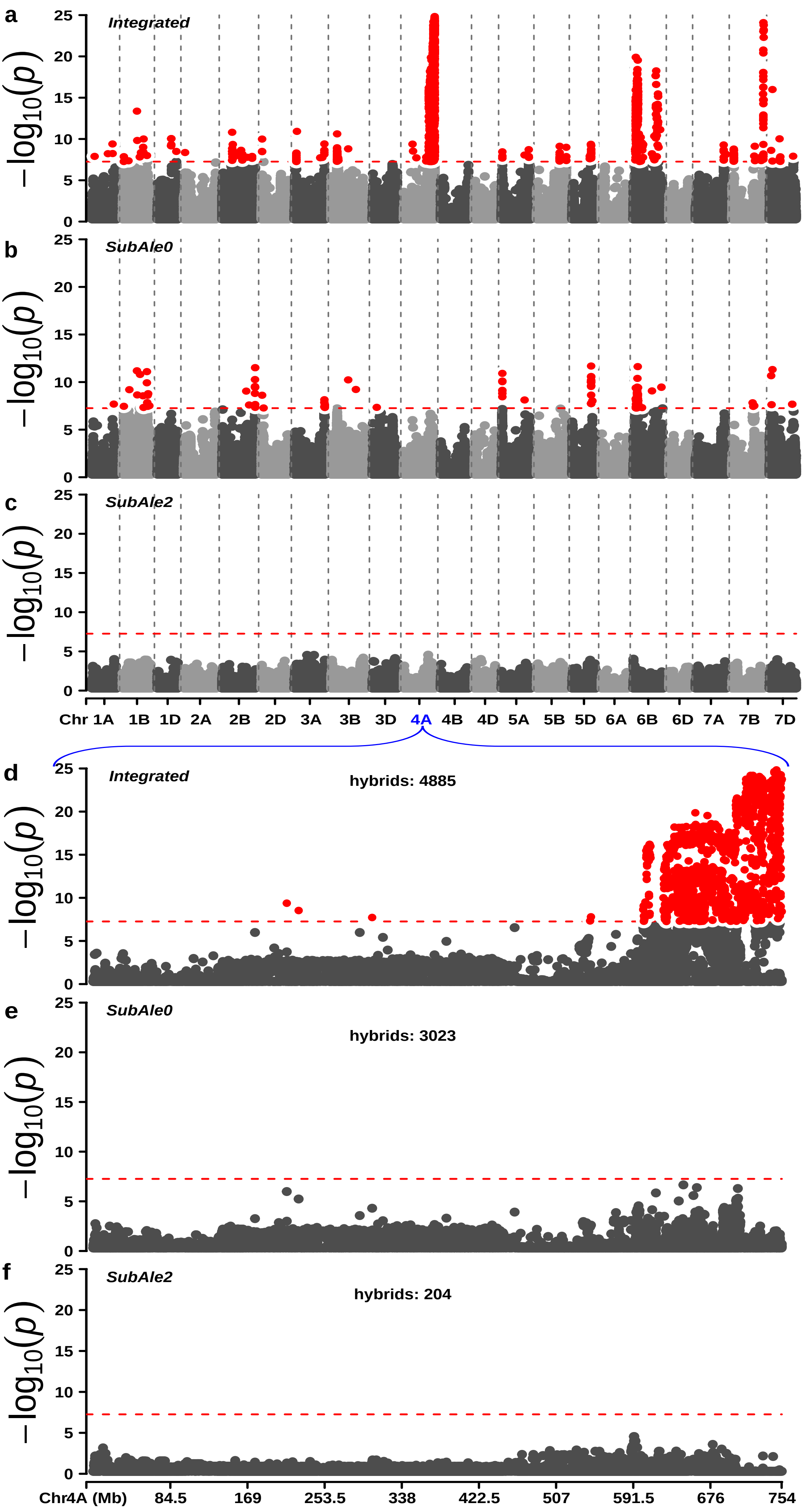

a
Integrated
b
SubAle0
c
SubAle2
Chr 1A 1B 1D 2A 2B 2D 3A 3B 3D 4A 4B 4D 5A 5B 5D 6A 6B 6D 7A 7B 7D
d
Integrated
hybrids: 4885
e
SubAle0
hybrids: 3023
f
SubAle2
hybrids: 204
Chr4A (Mb) 84.5 169 253.5 338 422.5 507 591.5 676 754
−log10(p)
0 5 10 15 20 25
