# Supplementary material for: Genetic Dissection of Wheat Heterosis Identifies Candidate Genes in Biparental and Diverse Hybrid Populations
Source: Plant Biotechnol J. 2026 Aug 30;24(11):5848–64. doi: 10.1111/pbi.70748 (PMC13527459; doi:10.1111/pbi.70748)
Supplement: Supplementary file 1 [file PBI-24-5848-s002.zip › pbi70748-sup-0008-FigureS7@Figure S7.pdf]

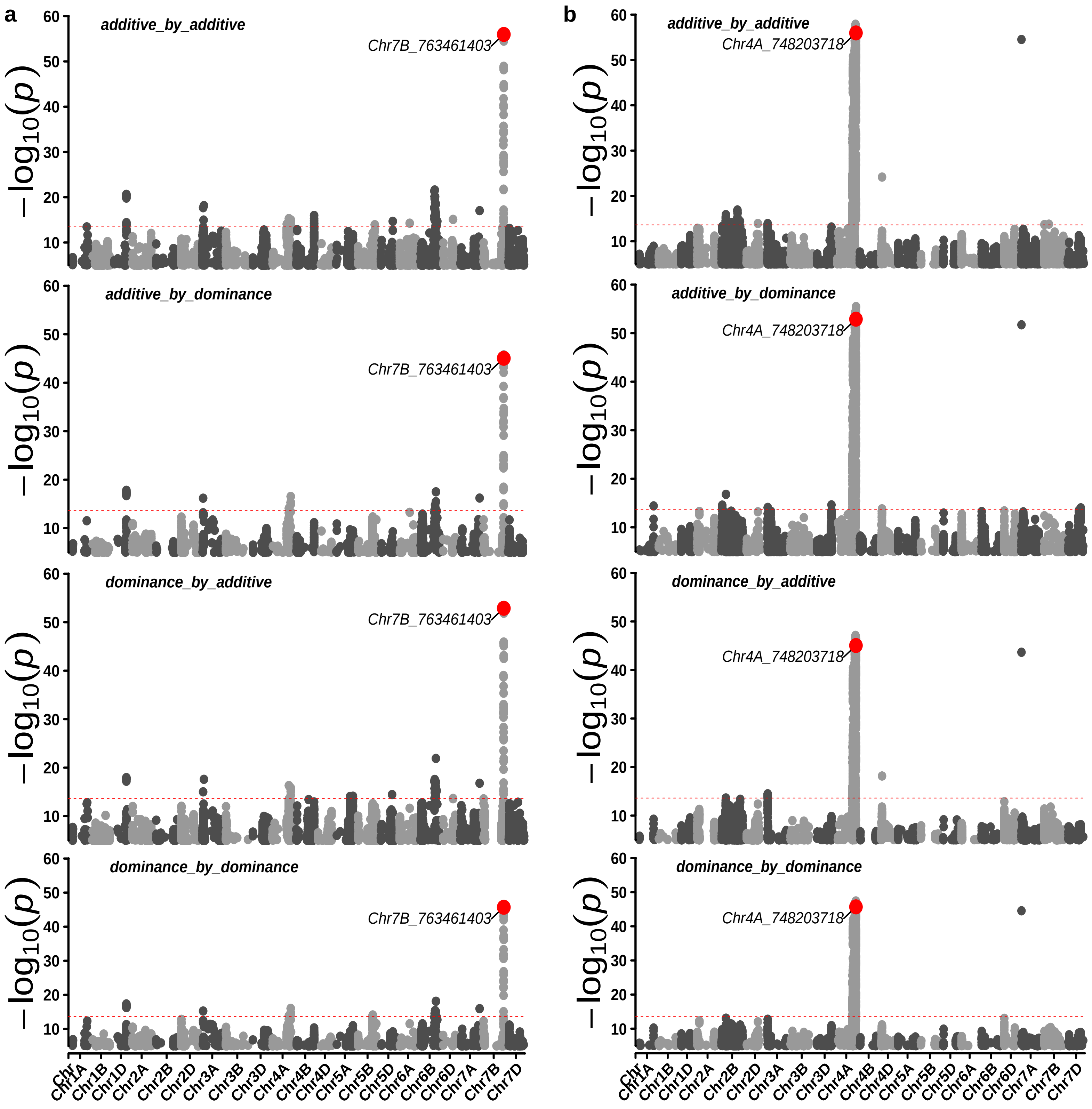

a
b
additive_by_additive
additive_by_dominance
dominance_by_additive
dominance_by_dominance
Chr7B_763461403
Chr4A_748203718
$-\log_{10}(p)$
Chr
Chr1A
Chr1B
Chr1D
Chr2A
Chr2B
Chr2D
Chr3A
Chr3B
Chr3D
Chr4A
Chr4B
Chr4D
Chr5A
Chr5B
Chr5D
Chr6A
Chr6B
Chr6D
Chr7A
Chr7B
Chr7D
