# Supplementary material for: Genetic Dissection of Wheat Heterosis Identifies Candidate Genes in Biparental and Diverse Hybrid Populations
Source: Plant Biotechnol J. 2026 Aug 30;24(11):5848–64. doi: 10.1111/pbi.70748 (PMC13527459; doi:10.1111/pbi.70748)
Supplement: Supplementary file 1 [file PBI-24-5848-s002.zip › pbi70748-sup-0009-FigureS8@Figure S8.pdf]

Define hQTL_CGR intervals based on hQTL identified in each experimental series using LD-pruned SNPs

hQTL	HD	PH	GY
Exp I	62	345	161
Exp II	293	53	66
Exp III	35	295	76
co-localization			
**hQTL_CGR**	**16 CGR**	**28 CGR**	**15 CGR**

Retrieve all SNPs within the hQTL_CGR intervals, including both LD-pruned SNPs and SNPs removed during LD pruning

Panel	SNP number	HD (16 CGR)	PH (28 CGR)	GY (15 CGR)
Exp I	LD pruning	31,841	41,888	28,691
	**without LD pruning**	**552,953**	**783,642**	**610,775**
Exp II	LD pruning	16,860	21,770	11,973
	**without LD pruning**	**134,690**	**160,170**	**97,497**
Exp III	LD pruning	9,618	13,289	8,634
	**without LD pruning**	**48,934**	**64,890**	**46,944**

hQTL scanning for all SNPs within the CGR intervals at two hierarchical levels

Exp-level (3)
Experimental series I
Experimental series I
Experimental series II

Env-level (137 environments)

Env. No	HD	PH	GY
Exp I	10	14	11
Exp II	20	21	12
Exp III	18	19	12

Nominate candidate genes harboring repeatedly detected heterotic SNPs with potentially functional variants

For GY heterosis:
TraesCS7B03G1341000

For GY heterosis:
(1) Chr4A: 24 candidate genes
(2) Chr7B: *TraesCS7B03G1340100* and ***TraesCS7B03G1341000***
