# Supplementary material for: Genetic Dissection of Wheat Heterosis Identifies Candidate Genes in Biparental and Diverse Hybrid Populations
Source: Plant Biotechnol J. 2026 Aug 30;24(11):5848–64. doi: 10.1111/pbi.70748 (PMC13527459; doi:10.1111/pbi.70748)
Supplement: Supplementary file 1 [file PBI-24-5848-s002.zip › pbi70748-sup-0010-FigureS9@Figure S9.pdf]

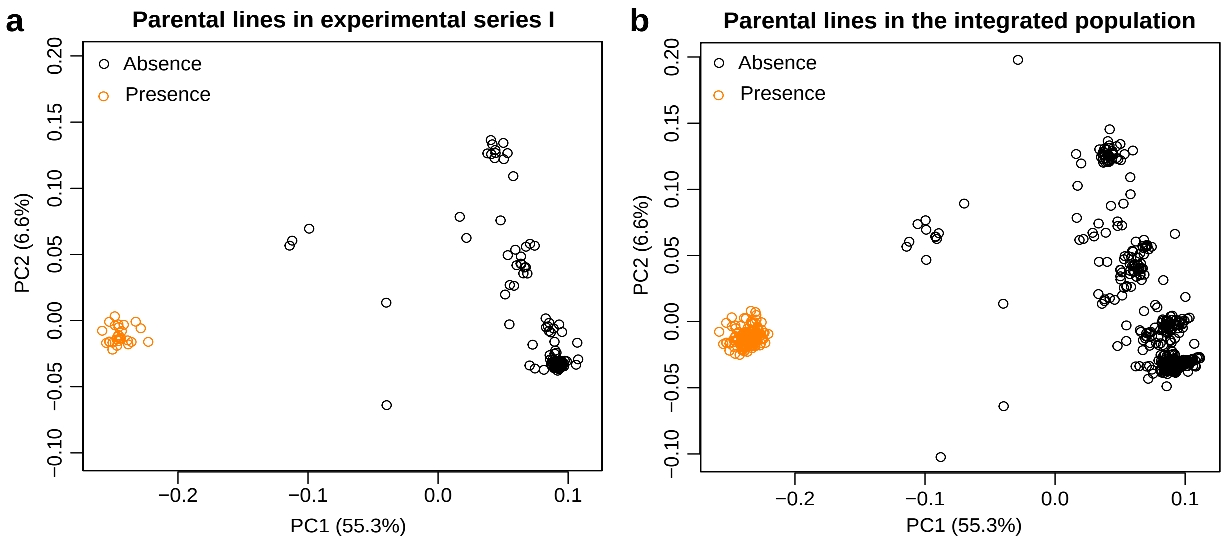
a
Parental lines in experimental series I
Absence
Presence
PC2 (6.6%)
PC1 (55.3%)
b
Parental lines in the integrated population
Absence
Presence
PC2 (6.6%)
PC1 (55.3%)
