# Supplementary material for: Genetic Dissection of Wheat Heterosis Identifies Candidate Genes in Biparental and Diverse Hybrid Populations
Source: Plant Biotechnol J. 2026 Aug 30;24(11):5848–64. doi: 10.1111/pbi.70748 (PMC13527459; doi:10.1111/pbi.70748)
Supplement: Supplementary file 1 [file PBI-24-5848-s002.zip › pbi70748-sup-0001-FigureS1@Figure S1.pdf]

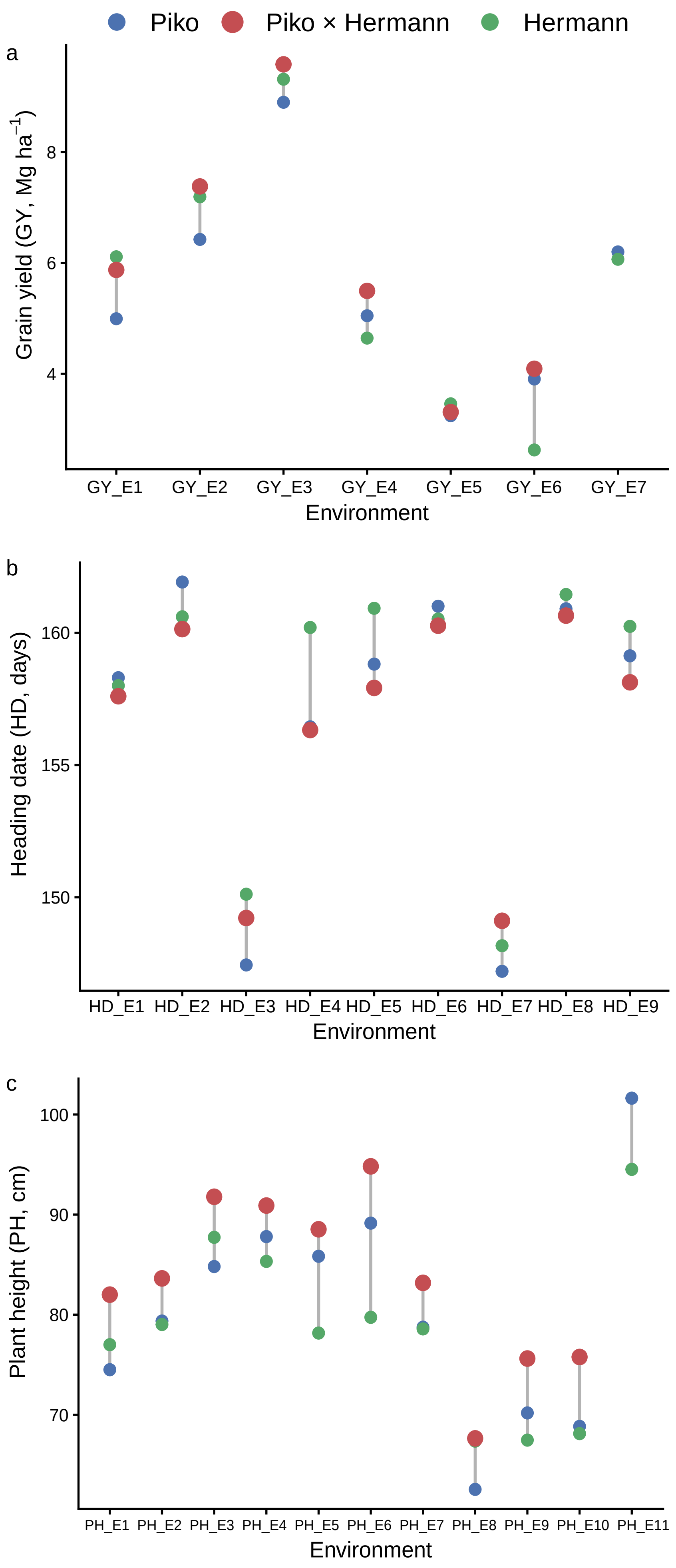
Piko
Piko × Hermann
Hermann
a
Grain yield (GY, Mg ha^{-1})
8
6
4
GY_E1
GY_E2
GY_E3
GY_E4
GY_E5
GY_E6
GY_E7
Environment
b
Heading date (HD, days)
160
155
150
HD_E1
HD_E2
HD_E3
HD_E4
HD_E5
HD_E6
HD_E7
HD_E8
HD_E9
Environment
c
Plant height (PH, cm)
100
90
80
70
PH_E1
PH_E2
PH_E3
PH_E4
PH_E5
PH_E6
PH_E7
PH_E8
PH_E9
PH_E10
PH_E11
Environment
