# Supplementary material for: Genetic Dissection of Wheat Heterosis Identifies Candidate Genes in Biparental and Diverse Hybrid Populations
Source: Plant Biotechnol J. 2026 Aug 30;24(11):5848–64. doi: 10.1111/pbi.70748 (PMC13527459; doi:10.1111/pbi.70748)
Supplement: Supplementary file 1 [file PBI-24-5848-s002.zip › pbi70748-sup-0002-FigureS1-S9@WheatMPH_SuppInfo_R1_20260811.docx]

**Genetic dissection of wheat heterosis identifies candidate genes in biparental and diverse hybrid populations**

Guoliang Li^1^*, Jianting Chu^1^, Max Haupt^1^, Yong Jiang^1^*, Jochen C. Reif^1^*

^1^Leibniz Institute of Plant Genetics and Crop Plant Research (IPK) OT Gatersleben, Corrensstr 3, 06466 Seeland, Germany

*Correspondence: Jochen C. Reif ([reif@ipk-gatersleben.de](mailto:reif@ipk-gatersleben.de)), Yong Jiang ([jiang@ipk-gatersleben.de](mailto:jiang@ipk-gatersleben.de)), and Guoliang Li ([lig@ipk-gatersleben.de](mailto:lig@ipk-gatersleben.de))

**Supporting Information**

Supplementary Figure S1-S9

Supplementary Notes

**Figure S1.** Grain yield performance of the Piko × Hermann hybrid and its parental lines across environments.

**Figure S2.** Genetic architecture of midparent heterosis for heading date (days) in the hybrid Piko × Hermann. (a) The genome wide scan of hQTL within the TTC population. (b) A genome-wide scan of the dominance was conducted within IMF2 population.

**Figure S3.** Schematic illustration of whole-genome re-sequencing data curation, GWAS mapping analysis and whole genome-wide prediction of MPH.

**Figure S4.** Manhattan and QQ plot for heterotic effects and dominance effects in the integrated population. (a, c, e) Plot for heterotic effect of grain yield, heading date, and plant height. (b, d, f) Plot for dominance effect of grain yield, heading date, and plant height.

**Figure S5.** Heatmap of linkage disequilibrium (LD) between all SNPs from the distal end of chromosome 4A, spanning the region from 602.37 Mb to 754.16 Mb.

**Figure S6.** Genome-wide Manhattan plots of heterotic effect in the integrated population (a) and in the two subpopulation classified by SNP Chr4A_748203718, 0 means without introgression (b) and 2 means with introgression (c). And (d–f) show enlarged views of the chromosome 4A region for the integrated population (d), the subpopulation without the Chr4A introgression (e), and the subpopulation with the Chr4A introgression (f).

**Figure S7.** Manhattan plot of epistatic interaction between significant SNPs (Chr4A_748203718 (a) and Chr7B_763461403 (b)) with the remaining genomic background for grain yield.

**Figure S8.** Schematic overview of the data-driven approach for candidate gene prioritization by retrieving all SNPs within hQTL regions and integrating association analyses from individual environments within each experimental series.

**Figure S9.** Principal coordinate analysis (PCoA) plots of the first two components for parental lines from experimental series I (a) and Integrated population (b) based on the Rogers’ distance matrix calculated using SNPs located in the distal end of chromosome 4A introgression genomic region (from Chr4A: 732,979,379 to 754,227,511).

**Supplementary Notes**

**Phenotypic and genotypic data in the biparental population of *Piko* × *Hermann***

*Phenotypic data analysis.* The following linear mixed model was used for phenotypic data analysis across environments in a one-step procedure.

Phenotype ~ Group + Genotype + Env + Genotype × Env + Rep(Env) + Block(Rep(Env)) + residual (1)

Where the “Group” effect included specific means of DH, TCP, TCH, TCF and IMF_2_ populations, “Genotype”, “Genotype × Env”, “Env,” “Rep,” and “Block” were the effects of genotypes, genotype-by-environment interactions, environments, replications, and blocks, respectively. Brackets indicate that the effects were nested within the other factor, e.g., “Rep(Env)” means that the effect of replications were nested within environments. All effects except the group effect were treated as random. The variance components were estimated by the REML method using the software ASReml-R 4.0 (Butler *et al.*, 2018), and the significance of the variance component estimates were tested by model comparison with likelihood ratio test (Anscombe and Tukey, 1963). The broad-sense heritability was calculated as $H^{2}={\sigma_{G}^{2}}/{(\sigma_{G}^{2}+{\sigma_{G\times E}^{2}}/{nEnv}+{\sigma_{e}^{2}}/{(nEnv\times nRep)})}$, where $nEnv$ refers to the number of environments, $nRep$ is the number of replications, $\sigma_{G}^{2}$, $\sigma_{G\times E}^{2}$ and $\sigma_{e}^{2}$ are the variance components of genotypes, genotype-by-environment interactions and residuals, respectively. In addition, the model was fitted once again assuming fixed genotypic effects to obtain BLUEs of all genotypes.

*Genotypic data.* The genotypic data of *Piko*, *Hermann* and all DH lines were obtained by merging the SNP chip array markers and genotyping by sequencing (GBS) profiles, as described in previous studies (Zapata *et al.*, 2025). Briefly, all lines in base population were genotyped with the optimized wheat 25K Infinium iSelect array containing 24,145 SNPs (SGS Institute Fresenius GmbH TraitGenetics Section, Gatersleben, Germany). GBS was performed on a single leaf harvested from a 2-week-old seedling for all genotypes. After removing adaptors and low-quality reads, high-quality reads were aligned against the wheat reference genome (Chinese Spring, RefSeq v2.1). In total, 10,881 high-quality SNPs were obtained by combing markers from the two genotyping platforms (Zapata *et al.*, 2025). Accompanied by the inspection of graphical genotypes, manually curated linkage maps were constructed for the DH population. Since many of the markers showed co-segregation in the biparental population, a bin map comprised of 743 representative markers was developed for subsequent QTL mapping. The high quality of the linkage map was confirmed by the fact that the order of markers on the linkage groups was in very good agreement with the order on the chromosome sequence maps (Chinese Spring, RefSeq v2.1) (Zapata *et al.*, 2025). Missing genotypes were imputed using the software Beagle version 5.2 (Browning *et al.*, 2018).

**Phenotypic and whole-genome sequencing data for the diverse hybrid wheat population**

*Field trials*. A total of 597 wheat elite parental lines and 5,243 F_1_ hybrid progenies from three large hybrid wheat populations (Experimental series I, II and III, (Li *et al.*, 2025; Zhao *et al.*, 2021)) were utilized for this study. Materials were chosen to reflect a wide range of diversity in Central Europe hybrid breeding program. Details of experimental design and information of phenotyping for grain yield (Mg/ha) and heading date (days from January 1^st^) has been previously reported in (Li *et al.*, 2025). For plant height (cm), all materials in experimental series I (Exp I, 120 females, 15 males and their 1604 hybrid progenies) were evaluated in 14 environments (6 locations in 2012 and 8 in 2013) in Germany. Experimental series II (Exp II, 185 females, 41 males, and their 1815 hybrids progenies) were evaluated in 21 environments in Germany (11 locations in 2016 and 10 in 2017). Experimental series III (Exp. III, 196 females, 40 males, and their 1824 hybrid progenies) were evaluated in 19 environments (10 locations in 2018 and 9 in 2019) in Germany. Phenotypic evaluations were conducted under field conditions using plots. Grain yield was evaluated in plots ranging from 5.0 to 7.4 m² (Exp I), 5.7 to 10.0 m² (Exp II), and 5.7 to 9.0 m² (Exp III). The sowing density was 200 seeds m⁻². For convenience, a summary of phenotyping information for all three traits was listed in the Table S5.

Curation and analysis of phenotypic data for plant height were performed by using the same procedure of analyzing grain yield and heading date data as described in (Li *et al.*, 2025). Briefly, a two-step approach was applied first within each experimental series and then across them. In the first step, phenotypic data within each environment were analyzed by fitting a linear mixed model (LMM) including the effects of genotypes, trials, replications nested with trials, and blocks nested within trials and replications. Best linear unbiased estimations (BLUEs) of the genotypes in each environment were obtained and served as the input for the second step, where a linear mixed model was implemented by including the effects of genotypes (parental lines and hybrids were separately fitted by using a dummy variable), environments and experimental series (only for the analysis across experimental series). The variance components and broad-sense heritability were estimated by treating all effects as random, while the BLUEs of genotypes across environments were obtained by setting the genotype effects as fixed variables. The heritability of MPH was estimated in a similar way. For convenience, the results of phenotypic data and MPH analysis for all three traits were summarized in Table S5.

*Whole-genome sequencing data.* All parental lines in this study were genotyped using the whole-genome resequencing (WGS) technology as previously described in details (Li *et al.*, 2025). Briefly, after removing adaptors and low-quality reads, 172.8 billion high-quality paired-end reads from all genotypes were aligned against the wheat reference genome (Chinese Spring, RefSeq v2.1). Ultimately, a total of 7,835,467 SNPs with minor allele frequency (MAF) ≥ 5% remained for the integrated population. After linkage disequilibrium (LD) pruning, 907,534 SNPs remained and were used for hQTL scanning (Figure S3). LD between all SNPs was calculated. For each SNP, if the number of SNPs on other chromosomes with LD values greater than 0.7 exceeded the number on its own chromosome, the SNP was considered likely to be mis-assigned due to ambiguous chromosome assignment. Such SNPs in the integrated population, totaling 19,897 from the full dataset and 5,218 from the pruned dataset (Figure S3), were excluded from further analyses. The corresponding numbers were 42,598 and 11,245 in experimental series I, 12,345 and 4,253 in experimental series II, and 8,250 and 2,878 in experimental series III (Figure S3). Compared with our previous study (Li *et al.*, 2025), this additional LD-based filtering step represented a major source of the updated results presented in this study.

**The delineation of QTL**

Based on GWAS results, LD information between significant SNPs and their neighboring markers was used to determine independent QTL regions. The QTL delineation was conducted using all available SNPs, including those removed during LD pruning (Figure S3), to obtain a more precise estimate of local LD decay and QTL boundaries. This refinement represents a key methodological difference from the approach adopted by Li et al. (Li *et al.*, 2025). First, for each significant SNP, we defined its upstream and downstream LD boundaries by assessing its LD (measured as r²) with neighboring SNPs. Specifically, the QTL boundary was set at the nearest genomic position where the LD dropped below 0.3. This step yielded a unique LD-defined interval for each significant SNP. Next, LD-defined QTL intervals were merged into independent QTL regions in the following two cases: (1) intervals overlapped; (2) intervals did not overlap but met both of the following conditions: (2a) the distance between their lead SNPs was less than 10 Mb (determined by the average genome-wide LD decay), and (2b) the average LD between all SNPs in the two intervals exceeded 0.3. This merging process was applied iteratively until no further merging steps are possible. The final set of non-redundant QTL intervals was designated as independent QTL. Within each QTL, the SNP with the lowest p-value was defined as the lead SNP, and the proportion of phenotypic variance explained (PVE) by the lead SNP was assigned as the representative PVE for that QTL. The same strategy was used to identify independent epistatic pairs. Markers at both ends of each significant epistatic interaction were first assigned to their respective QTL intervals. Significant interactions were then consolidated at the QTL-interval level, and the number of independent epistatic pairs was determined by counting unique pairs of interacting QTL intervals.

**Data-driven prioritization of candidate genes**

To facilitate candidate gene identification with each hQTL, we first extracted all genes located within the respective QTL intervals (if interval size > 0) based on the Chinese Spring reference genome (CSv2.1; Zhu et al., 2021). For hQTL with zero interval size, namely hQTL consisting of a single SNP, the gene closest to the SNP was listed, and the physical distance from the SNP to this nearest gene was recorded in the summary table (Table S6-S28). To provide functional information for candidate gene evaluation, we incorporated multiple sources of gene annotations. Gene function annotations from the CSv2.1 genome was retained, and additional annotations were retrieved from the wheat Gene Regulatory Network database (wGRNv1, <http://wheat.cau.edu.cn/wGRN/>, (Chen *et al.*, 2023)). Furthermore, broader functional annotations were obtained from eggNOG-mapper (<http://eggnog-mapper.embl.de>), a tool that provides orthology-based functional classification. Variant annotation was performed by using ANNOVAR (Yang and Wang, 2015) and applying 1,000 bp windows to define upstream and downstream regions of genes. All significant SNPs, as well as all variants (including non-significant ones) located within each gene body and its flanking regions (1,000 bp upstream and downstream), were annotated and specifically highlighted the annotations of significant SNPs (Table S6–S25). Based on the nature of the variants and their statistical significance, genes were ranked into four priority levels: Level 1 includes genes harboring significant SNPs with potentially functional variants (i.e., nonsynonymous variants, splice donor/acceptor site variants, or start/stop-gain/loss mutations); Level 2 includes genes with significant SNPs but without functionally impactful variants; Level 3 includes genes without significant SNPs but with functional variants; Level 4 includes all remaining genes that harbor neither significant SNPs nor impactful variants.

To further refine causal gene identification, we implemented a data-driven cross-check approach (Figure S8) to systematically prioritize variants and genes at trait-associated loci. Beyond incorporating gene annotation information, this approach characterized retrieving all SNPs located within the target interval, including those previously excluded during LD pruning, and re-evaluating their significance at two hierarchical levels: among the three experimental series and among environments within each series. The target intervals were defined as hQTL co-localization regions detected in at least two experimental series that either overlapped or were located within 1 Mb of each other; these regions were subsequently combined into a single genomic region (hQTL_CGR) with an additional ±0.5 Mb extension. Using this criterion, we identified 16 hQTL_CGR for heading date heterosis, 28 hQTL_CGR for plant height heterosis, and 15 hQTL_CGR for grain yield heterosis (Figure S8). Ultimately, heterotic SNPs (hSNPs) that was repeatedly detected and predicted to have functional impacts were considered the most informative candidates. Accordingly, we summarized two categories of recurrent hSNPs: (i) cross-series recurrent hSNPs (cs-hSNPs), defined as hSNPs identified in all three experimental series; and (ii) multi-environment recurrent hSNPs (me-hSNPs), defined as hSNPs identified in at least two environments regardless of whether they occurred within or across experimental series.

**References**

Anscombe, F.J. and Tukey, J.W. (1963) *The Examination and Analysis of Residuals*. *Technometrics*, **5**, 141–160.

Browning, B.L., Zhou, Y., and Browning, S.R. (2018) *A One-Penny Imputed Genome from Next-Generation Reference Panels*. *The American Journal of Human Genetics*, **103**, 338–348.

Butler, D.G., Cullis, B.R., Gilmour, A.R., Gogel, B.J., and Thompson, R. (2018) *ASReml-R Reference Manual Version 4*. Hemel Hempstead, UK: VSN International Ltd.

Chen, Y., Guo, Y., Guan, P., Wang, Y., Wang, X., Wang, Z., et al. (2023) *A wheat integrative regulatory network from large-scale complementary functional datasets enables trait-associated gene discovery for crop improvement*. *Molecular Plant*, **16**, 393–414.

Li, G., Schmidt, R.H., Zhao, Y., Reif, J.C., and Jiang, Y. (2025) *Powerful one-dimensional scan to detect heterotic quantitative trait loci*. *Nat Commun*, **16**, 9697.

Zapata, S.L., Schulthess, A.W., El Hanafi, S., Schmidt, R.H., Hinterberger, V., Li, G., et al. (2025) *Disentangling the genetic architecture of key traits for wheat hybrid seed production*. *Journal of Experimental Botany*, **76**, 5320–5336.

Zhao, Y., Thorwarth, P., Jiang, Y., Philipp, N., Schulthess, A.W., Gils, M., et al. (2021) *Unlocking big data doubled the accuracy in predicting the grain yield in hybrid wheat*. *Sci. Adv.*, **7**, eabf9106.
